# Suitability of prolonged meloxicam treatment in mice seems limited due to unfavorable pharmacokinetics, side effects, and impact on home-cage behaviors

**DOI:** 10.1038/s41598-025-25180-4

**Published:** 2025-11-07

**Authors:** Aylina Glasenapp, Jens P. Bankstahl, Heike Bähre, Jana Hauser, Amisha R. Parmar, Andrey V. Kozlov, Silke Glage, Rupert Palme, Marion Bankstahl

**Affiliations:** 1 Institute for Laboratory Animal Science and Central Animal Facility, Hannover Medical School, Hannover, Germany; 2 Department of Nuclear Medicine, Hannover Medical School, Hannover, Germany; 3 Research Core Unit Metabolomics, Hannover Medical School, Hannover, Germany; 4 Department of Biological Sciences and Pathobiology, Center for Biological Sciences, Pharmacology and Toxicology, University of Veterinary Medicine Vienna, Vienna, Austria; 5 Ludwig Boltzmann Institute for Traumatology, The Research Center in Cooperation with AUVA, Austrian Cluster for Tissue Regeneration, Vienna, Austria; 6 Department of Biological Sciences and Pathobiology, Center for Biological Sciences, Experimental Endocrinology, University of Veterinary Medicine Vienna, Vienna, Austria

**Keywords:** Behavioural methods, Mouse, Mass spectrometry, Pharmacokinetics

## Abstract

Meloxicam is commonly used for analgesia, but no preparation approved specifically for use in mice. Here, we determined plasma concentrations, safety, and impact on home-cage behaviors for subcutaneous injection (5 mg/kg) and oral self-intake (sweetened drinking water, 20 mg/kg/24h, 5 days) for C57BL/6J mice (n = 21/sex). After injection, plasma concentrations measured by LC–MS/MS were higher in females (2h) and remained within an estimated therapeutic range (390–911 ng/mL) for up to 6h. T_1/2_ was 2.32 h. Despite acceptance, plasma levels fluctuated strongly during oral self-intake. Side effects comprised reduced grip strength and increased vocalization (Irwin test), increased clinical score values (females, oral treatment) with two individuals reaching humane endpoint, increase in body temperature, body weight drop, decreased wheel-running activity, increased burrowing latency (males), and prolonged grooming activity (females). Grimace scale, nesting activity and plasma corticosterone remained unaffected. Red blood profile parameters and ALT were decreased and Ca^2+^ and Cl^-^ concentrations elevated. Histopathological analysis revealed inflammatory cells and hyperplasia in stomach and jejunum. In view of the unfavorable pharmacokinetics, meloxicam treatment via the drinking water is unsuitable in mice. The limited tolerability make prolonged treatment with the doses studied appear inappropriate.

**Supplementary Information:**

The online version contains supplementary material available at 10.1038/s41598-025-25180-4.

## Introduction

Experimental studies in rodents frequently involve surgical interventions or disease states necessitating adequate pain relief. The non-steroidal anti-inflammatory drug (NSAID) meloxicam (MEL) is commonly used to attenuate mild to moderate pain both in veterinary practice and laboratory animals. Its mechanism of action is related to inhibition of prostaglandin synthesis by cyclooxygenases (COX), whereby MEL has been shown to selectively inhibit COX-2 over COX-1^1,2^. To the best of our knowledge, to date, no medicinal product containing meloxicam has been officially approved for use in mice (worldwide), and recommended doses range widely between 1 and ^3^ with an injection interval of 6–24 h^3–6^ despite a relatively short plasma elimination half-life (t_1/2_) of 2.22 h reported for young C57BL/6 mice for subcutaneous (s.c.) injection^7^. Also, data on analgesic efficacy remain inconclusive^3–6,8,9^. Besides s.c. injection, oral self-intake of analgesics from the drinking water (d.w.) might represent an alternative administration method due to its non-invasive nature. For carprofen as another frequently used NSAID, we have recently shown very good acceptance and stable plasma levels reached both in mice and rats during prolonged treatment via the d.w.^10,11^. MEL might be suitable for this approach as well as it is stable in water for at least 7 days, but attention has to be given to palatability, as the consumption of MEL-medicated water might be reduced in mice^12^. While species-specific data on pharmacokinetics (PK) become increasingly available for s.c. administration of MEL in mice^7,12–14^, data on oral consumption and related plasma concentrations as well as tolerability remain very limited^12,15^.

On the one hand, MEL is among the most frequently applied analgesics in mice^16^, but on the other hand evidence for side effects induced by prolonged use of high-dose MEL (20 mg/kg) is cumulating. Reports about gastric ulcerations or skin lesions after repeated s.c. injections^17,18^, noxious effects on several organs after oral administration^19^, and even mortality^14^, demonstrate the necessity to further examine the safety of MEL in mice in a structured and comprehensive manner, comprising blood and histological analyses as well as behavioral parameters. Regarding the latter, besides mouse grimace scale, home-cage behavior such as burrowing, nesting or wheel running activity as well as measurement of blood or fecal corticosterone levels are increasingly applied for assessing pain and the effectiveness of pain relief after surgical procedures^20–22^. While potential confounding effects of MEL on those indicators of pain have rarely been studied in mice, understanding this seems crucial to distinguish between behavioral changes caused by pain or caused by MEL therapy.

First aim of this study was to determine PK profiles for MEL both for s.c. injection (5 mg/kg) and oral self-intake via the d.w. (20 mg/kg/24 h) for 5 days in healthy mice. Second, we aimed to create a detailed side effect profile for the MEL dosages applied in healthy mice, including the possible impact of prolonged treatment on established behavioral parameters indicative of pain or distress, as well as blood parameters.

## Results

### Short elimination half-life after s.c. injection and circadian-dependent plasma concentrations during oral intake

**Fig. 1:**
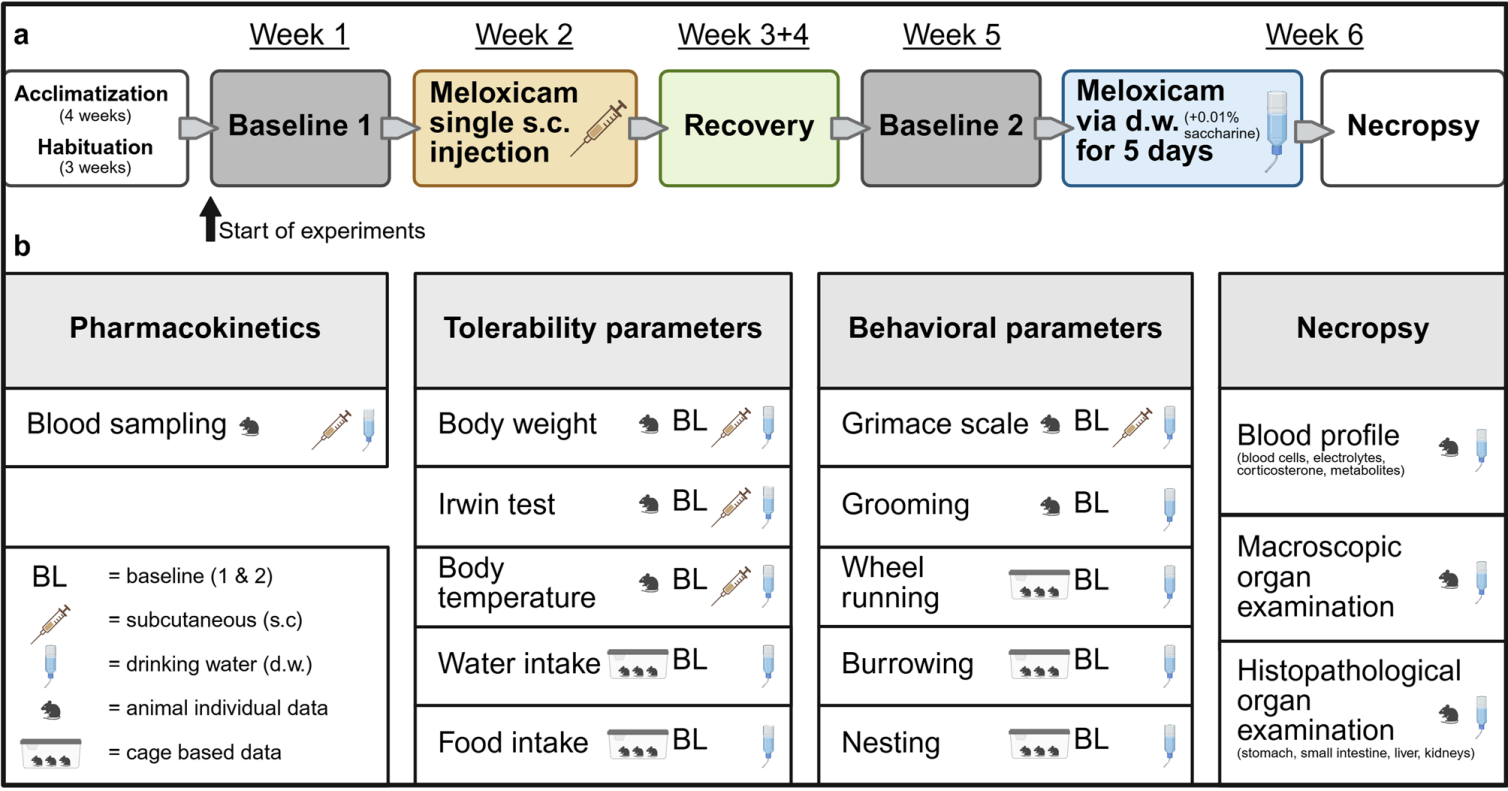
Experimental design. (**a**) Timeline of complete experimental setup. (**b**) Overview of all interventions and parameters. Created in BioRender. Bankstahl, M. (2025) https://BioRender.com/pe823wm.

**Fig. 2:**
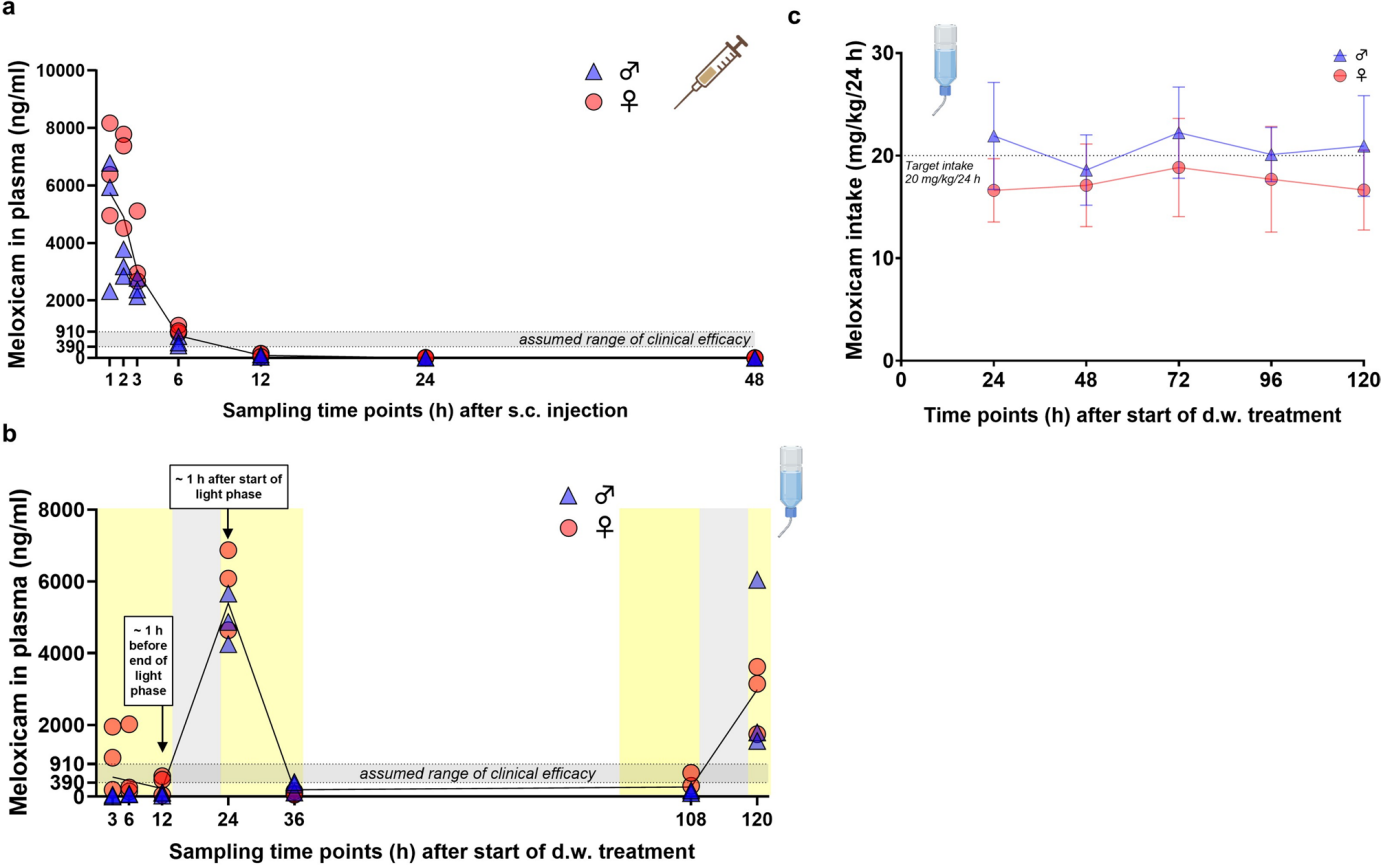
Plasma concentration–time curves following subcutaneous (s.c.) injection and during drinking water (d.w.) intake of meloxicam (MEL). (**a**) Plasma concentration–time curve following single s.c. injection of 5 mg/kg MEL. Individual data points (n = 6, 3 male and 3 female per time point) and mean of all animals per time point (black line) are presented. Assumed range of therapeutic plasma concentration (grey shaded area between 390 and 911 ng/mL) is displayed according to previously published data^23,24^. (**b**) Plasma concentration–time curve during oral intake of MEL (intended dose: 20 mg/kg/24 h) via d.w. over 5 consecutive days. Individual data points (n = 6, 3 male and 3 female per time point) and mean of all animals per time point (black line) are presented. Assumed range of clinical efficacy (horizontal grey shaded area between 390 and 911 ng/mL) is displayed according to previously published data^23,24^. Vertical yellow and gray background shading indicates the approximate duration of the light and dark phases. (**c**) MEL dose intake (mg/kg/24 h; calculated by consumption and concentration of MEL-medicated d.w.) is displayed for 24 h intervals after start of oral treatment (n = 7 cages per sex, 3 mice/cage). Target dose is indicated by dotted line.

An overview of the experimental setup is provided in Fig. 1. To generate PK profiles of MEL in mice, tail vein blood was collected at 1, 2, 3, 6, 12, 24, and 48 h after s.c. injection and at 3, 6, 12, 24, 36, 108, and 120 h after start of oral self-administration via sweetened d.w.. Quantification of MEL in plasma revealed C_max_ of 6.640 ± 1.610 ng/mL after 1 h and t_1/2_ of 2.32 h after single s.c. injection of 5 mg/kg MEL (Fig. 2a). Further PK parameters are given in Table 1. Plasma concentrations were above or within an assumed range of therapeutically useful levels of about 390–911 ng/mL (IC_50_ 390 ng/mL, analgesic effect in a paw inflammation model in dogs^23^; IC_50_ 911 ± 189 ng/mL, lameness score in cats^24^) for up to 6 h after injection. Oral self-administration of MEL (20 mg/kg/24 h) started in the morning of the light phase and resulted in C_max_ of 5.394.0 ± 986.9 ng/mL after 24 h (Fig. 2b). Plasma concentrations fluctuated greatly and were mainly only within or above the estimated therapeutic range at the end of the dark phases, i.e. when blood had been sampled in the mornings (beginning of light phase; 24, 120 h). Analysis of plasma sampled during (3, 6 h) or prior to the end of light phase (12, 36, 108 h) revealed MEL concentrations below the estimated therapeutic range in the majority of mice (except for 2 females at 3 h and 1 female at 6 h after start of d.w. administration). Sex differences were noted for plasma levels at 2 h after s.c. injection (females: 18.667 ± 5.700 ng/mL; males: 9.300 ± 1.343 ng/mL; p = 0.0274) but not during d.w. treatment.

**Table 1:** Noncompartmental analysis of pharmacokinetic parameters after subcutaneous injection of 5 mg/kg meloxicam in mice (n = 21 male, 21 female).

Parameter	Unit	Mean	SD
C_max_	ng/mL	6,640.0	1,610.0
T_max_	h	1.50	0.55
T_1/2_	h	2.32	0.28
AUC 0 → ∞	h/ng/mL	21,080.0	5,430.0
AUMC 0 → ∞	h^2^/ng/mL	67,140.0	18,190.0
MRT 0 → ∞	h	3.19	0.29
Vd/F	mL/kg	845.76	263.62
CL/F	mL/h/kg	251.52	67.76

C_max_, maximum plasma concentration; T_max_, time of maximum plasma concentration; T_1/2_, plasma elimination half-life; AUC 0 → ∞ , area under the curve from time 0 extrapolated to infinity; AUMC 0 → ∞ , area under the first moment curve from time 0 extrapolated to infinity; MRT 0 → ∞ , mean residence time from time 0 extrapolated to infinity; Vd/F, volume of distribution; CL/F, clearance.

Water consumption during the first 24 h after start of oral treatment revealed gradual incline of MEL intake (3 h: 3.5 ± 0.7 mg/kg; 6 h: 6.1 ± 1.6 mg/kg; 12 h: 10.6 ± 2.1 mg/kg, 24 h 19.3 ± 5.0 mg/kg; Supplementary Fig. S1a). During the first 48 h, in average 60% of total MEL intake was consumed during the dark phase (Supplementary Fig. S1b). Females consumed slightly less of the sweetened MEL-medicated water than targeted (17.4 ± 4.1 mg/kg/24 h; Fig. 2c), and fluid consumption was significantly reduced at 24 h after start of d.w. treatment compared to BL2 (*p* = 0.0010; Supplementary Fig. S3a). Male mice, however, accepted the sweetened MEL-medicated water well resulting in an average dose intake of 20.8 ± 4.2 mg/kg/24 h MEL over 5 days (Fig. 2c).

### Impact on Irwin test parameters, body temperature, body weight, clinical score as well as food and water intake particularly during prolonged oral self-intake of MEL

**Fig. 3:**
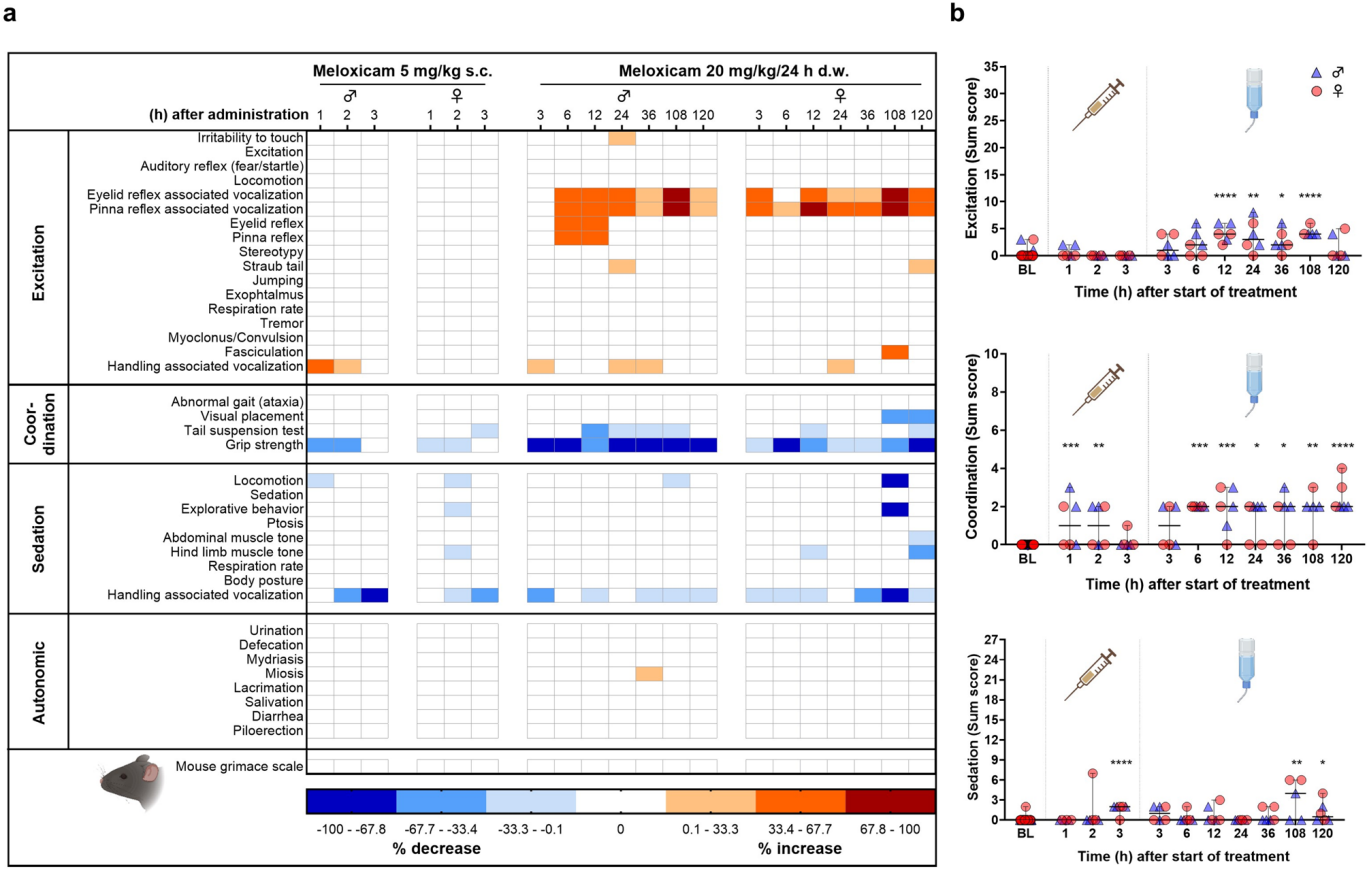
Tolerability of meloxicam (MEL) as assessed by modified Irwin test. (**a**) Impact of acute (single subcutaneous (s.c.) injection, 5 mg/kg) and prolonged (drinking water (d.w.), 5 days, 20 mg/kg/24 h) MEL administration on behavioral parameters and mouse grimace scale are displayed as heat map. Per time point and sex, 3 mice were investigated. Increase or decrease is presented as a percentage of the change in the total number of animals per sex and timepoint. (**b**) Kruskal–Wallis test followed by Dunn’s multiple comparisons test show significantly higher sum score values compared to baselne (BL) in the categories excitation, coordination, and sedation (*p < 0.05; **p < 0.01; ***p < 0.001; ****p < 0.0001).

Tolerability was evaluated by a modified Irwin test protocol covering a variety of read-out parameters^25^. These were categorized in excitation, coordination, sedation, autonomic symptoms, and mouse grimace scale, and displayed in a heat map (Fig. 3a). After s.c. injection, increased handling-associated vocalization was observed in male mice within the first 2 h, whereas decreased handling-associated vocalization was also noted in both sexes 2 and 3 h after injection. In addition, reduced grip strength was observed in both sexes up to 2 h. During oral intake of MEL via d.w., both sexes expressed a clear increase in reflex testing-associated vocalization (eyelid, pinna). Coordinative behavior was influenced in terms of reduced visual placement and impaired struggle during tail suspension test at some time points. At all investigated time points during MEL intake via d.w., grip strength was considerably reduced. Live scoring of the mouse grimace scale^26^ was performed as part of Irwin test procedure in the home cage and did not reveal increased score values at any time point. For the categories excitation, coordination, and sedation, a sum score for each individual animal and time point is presented in Fig. 3b. After s.c. injection, increased score values for coordination and sedation were observed, whereas during d.w. treatment significant changes compared to baseline were present in all three categories, characterized by increased excitation, reduced coordination (particularly grip strength), and slightly sedative effects (Fig. 3b).

**Fig. 4:**
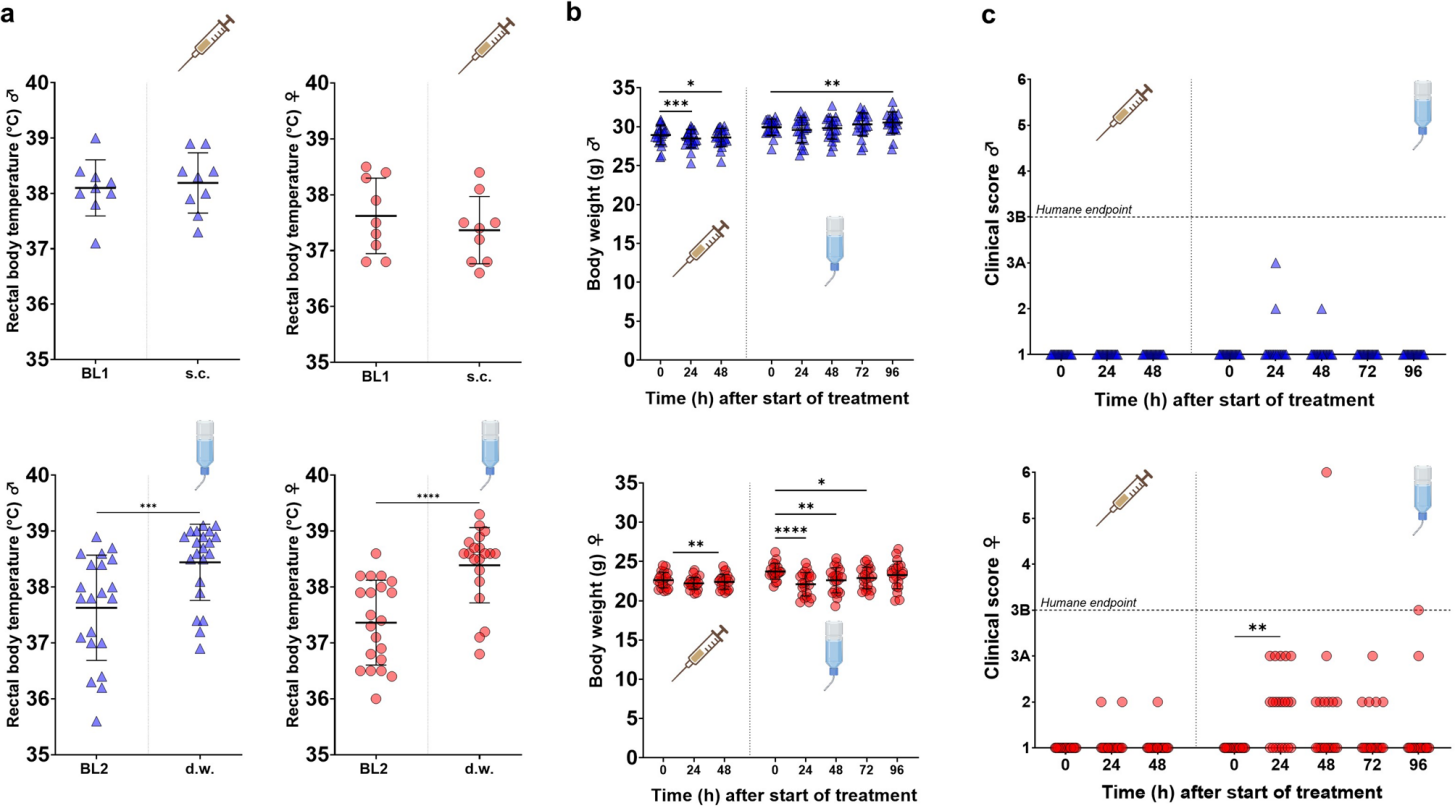
Meloxicam (MEL) intake via drinking water (d.w.) influences body temperature, body weight and clinical score in female mice. (**a**) Data from all measurements after subcutaneous (s.c.) MEL (5 mg/kg) treatment (upper graphs) compared to baseline 1 (BL1) and oral MEL intake (20 mg/kg/24 h) via d.w. (lower graphs) compared to baseline 2 (BL2) are shown. Wilcoxon test compared paired data after s.c. (n = 18; 9 male, 9 female) and d.w. (n = 42; 21 male, 21 female) treatment and shows increased body temperature during d.w. treatment, but not after s.c. injection of MEL compared to animal individual BL. Mean ± SD is shown. (**b**) Daily body weight of male (upper graph) and female (lower graph) mice throughout the experimental course is presented. Data points represent individual mice (n = 21 per sex), mean ± SD is shown. (**c**) Clinical score was assessed daily during s.c. and d.w. treatment. Male mice (2/21) showed transient increased clinical scores during oral MEL treatment (upper graph). (**c**) After s.c. injection, 3/21 female mice and during d.w. treatment 15/21 female mice were assessed with increased clinical scores. At 48 and 96 h after start of d.w. treatment, one female mouse each reached humane endpoint, respectively, was humanely killed. (*p < 0.05; **p < 0.01; ***p < 0.001; ****p < 0.0001).

Measurement of rectal body temperature immediately after Irwin test procedure revealed a mean temperature increase of around 0.8 °C in males and 1.0 °C in females during oral intake of MEL (*p* < 0.0001) but not after s.c. injection (Fig. 4a). Body weight dropped significantly in both sexes after s.c. injection (males: 24 h *p* < 0.0001, 48 h *p* = 0.0180; females: 24 h *p* = 0.0070) (Fig. 4b). In male mice, body weight loss remained below 5% in all individuals, whereas 3/21 female mice lost between 5 and 10% of body weight. During oral treatment, an increase in body weight at 96 h after treatment start was found in male mice, whereas body weight of females was significantly reduced on the first three days after start of MEL medication (24 h *p* < 0.0001, 48 h *p* = 0.0014, 72 h *p* = 0.0104). In total, 2/21 male and 8/21 female mice lost 5—10% of body weight, moreover 3/21 females lost 10—15% and 3/21 females lost 15—20% of their body weight during the d.w. treatment period.

Parallel to body weight measurements, mice underwent daily clinical scoring up to 48 h after s.c. injection and blood sampling and until the end of oral treatment according to a predefined scheme (Supplementary Fig. S2). After s.c. injection, male mice did not express abnormal clinical signs, whereas 3/21 female mice had transiently increased clinical score values (Fig. 4c). During d.w. treatment, both male (2/21) and especially female (14/21) mice were assessed with increased clinical score values (Fig. 4c), which were mainly influenced by body weight loss and mild changes in activity. This included two females reaching humane endpoint. One of these was found moribund with closed eyes, flat breathing, and in lateral position laying in the cage and therefore scored with the highest value (score 6) at 48 h after start of d.w. treatment and immediately killed humanely. The other mouse reaching humane endpoint scored 3B at 96 h under MEL treatment seemed clinically normal, but lost 16% of body weight since treatment start and did not recover despite parental liquid substitution. During necropsy, the caecum of this individual mouse was small and the colon completely empty. Clinical score values were significantly increased for females on group level at 24 h after start of oral MEL treatment (*p* = 0.0031). The deterioration of condition was acute and rapid without previously observed clinical signs. Accordingly, due to body weight loss and reaching score 3B, another female mouse was humanely killed at 96 h after start of d.w. treatment. Here, no abnormalities were observed during postmortem macroscopic organ examination.

Food consumption during oral MEL treatment was comparable to BL2 in both sexes, except for increased consumption at 48 h (BL2 12.87 ± 1.42 g/24 h vs MEL 17.17 ± 2.36 g/24 h, *p* = 0.0028) in female mice and a consumption drop at 120 h after start of treatment (BL2 16.79 ± 1.09 g/24 h vs MEL 12.66 ± 3.89 g/24 h,   *p* = 0.0133) in male mice (Supplementary Fig. S3b).

### Prolonged oral self-intake of MEL results in transient reduction of voluntary wheel running activity and limited effects on burrowing and grooming activity

**Fig. 5:**
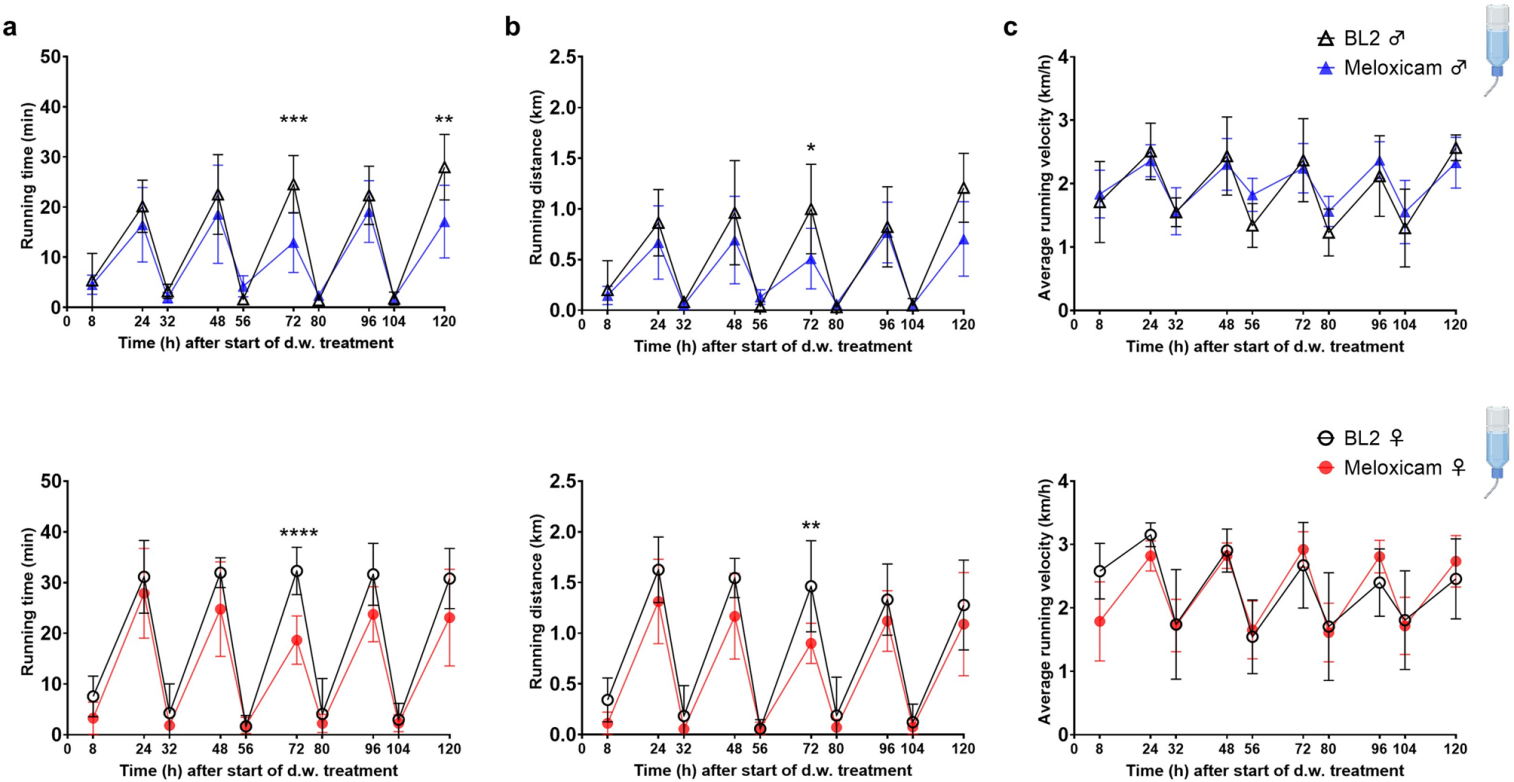
Meloxicam (MEL) treatment via drinking water (d.w.) is associated with slightly decreased wheel running activity in mice. (**a**) Running time (min), (**b**) running distance (km) and (**c**) average running velocity (km/h) is shown normalized to total time (h) during read out intervals (8 h during day and 16 h during night) for male (upper graphs) and female (lower graphs) mice. Running wheel data were documented between 8 and 9 am in the morning and between 4 and 6 pm in the afternoon. Data are shown as mean ± SD (n = 7 cages per sex, 3 mice/cage). Two-way ANOVA and Šídák’s multiple comparisons test reveals decrease in wheel running time and distance in mice at singular timepoints under MEL (20 mg/kg/24 h) treatment (*p < 0.05; **p < 0.01; ***p < 0.001; ****p < 0.0001). BL2, baseline 2.

Home-cage based behavioral parameters were investigated exclusively during oral MEL administration. During MEL d.w. treatment, wheel running activity was measured over five consecutive days during “dark” and “light” phases to detect potential influence of treatment on voluntary exercise (Fig. 5). Running time (min) overnight was decreased at 72 h after start of treatment in both sexes (− 47%, *p* = 0.0004 males; − 42%, *p* < 0.0001 females) and at 120 h in males (− 39%, *p* = 0.0070) vs. respective time points during BL2 (Fig. 5a). Running distance (km) was also decreased after 72 h in both sexes (− 49%, *p* = 0.0148 males; 39%, *p* = 0.0055 females) (Fig. 5b). For both sexes, running velocity was not influenced by MEL treatment (Fig. 5c). During the light phase, none of the wheel running parameters were affected.

During MEL administration via d.w., burrowing performance was assessed every day (in 24 h intervals) after start of treatment (Supplementary Fig. S4a). During the burrowing time of 2 h, male mice burrowed continuously stable amounts of pellets both at baseline assessments and during analgesic administration, but reduced their latency to start burrowing at 96 h (BL2, 11.86 ± 11.08 min; MEL, 3.14 ± 3.53 min; *p* = 0.0039). For female mice, a high variety in burrowing activity (latency and amount burrowed) was observed, and some cages did not burrow at all both during baseline phases and MEL administration via d.w..

Nesting behavior was assessed over 5 consecutive days twice per day using a modified established nest score^10,27^. Male mice underwent a first trial for 72 h (indicated as Meloxicam I ♂) and, after provision of new nesting material, a second trial for a duration of 38 h (indicated as Meloxicam II ♂) (Supplementary Fig. S4b). No statistically significant differences compared to BL2 were observed in nest building during oral MEL treatment for both sexes.

Grooming activity was assessed according to an established scoring protocol^27^ at 2, 14, and 38 h after application of fluorescent oil to the neck region (Supplementary Fig. S4c). Grooming test started by application of fluorescent oil at 10 h after start of MEL d.w. treatment, which was repeated at 58 h after treatment start. Grooming activity of females was decelerated at 72 h under MEL treatment (*p* = 0.01), and the necessary time to achieve score 5 was delayed (*p* = 0.0054) after the second application of fluorescent oil during MEL treatment (Supplementary Fig. S4d). In male mice, no changes in grooming behavior associated with MEL treatment were observed.

### Prolonged oral self-intake of MEL leads to inflammatory cell infiltration and mild hyperplasia in stomach and jejunum

**Fig. 6:**
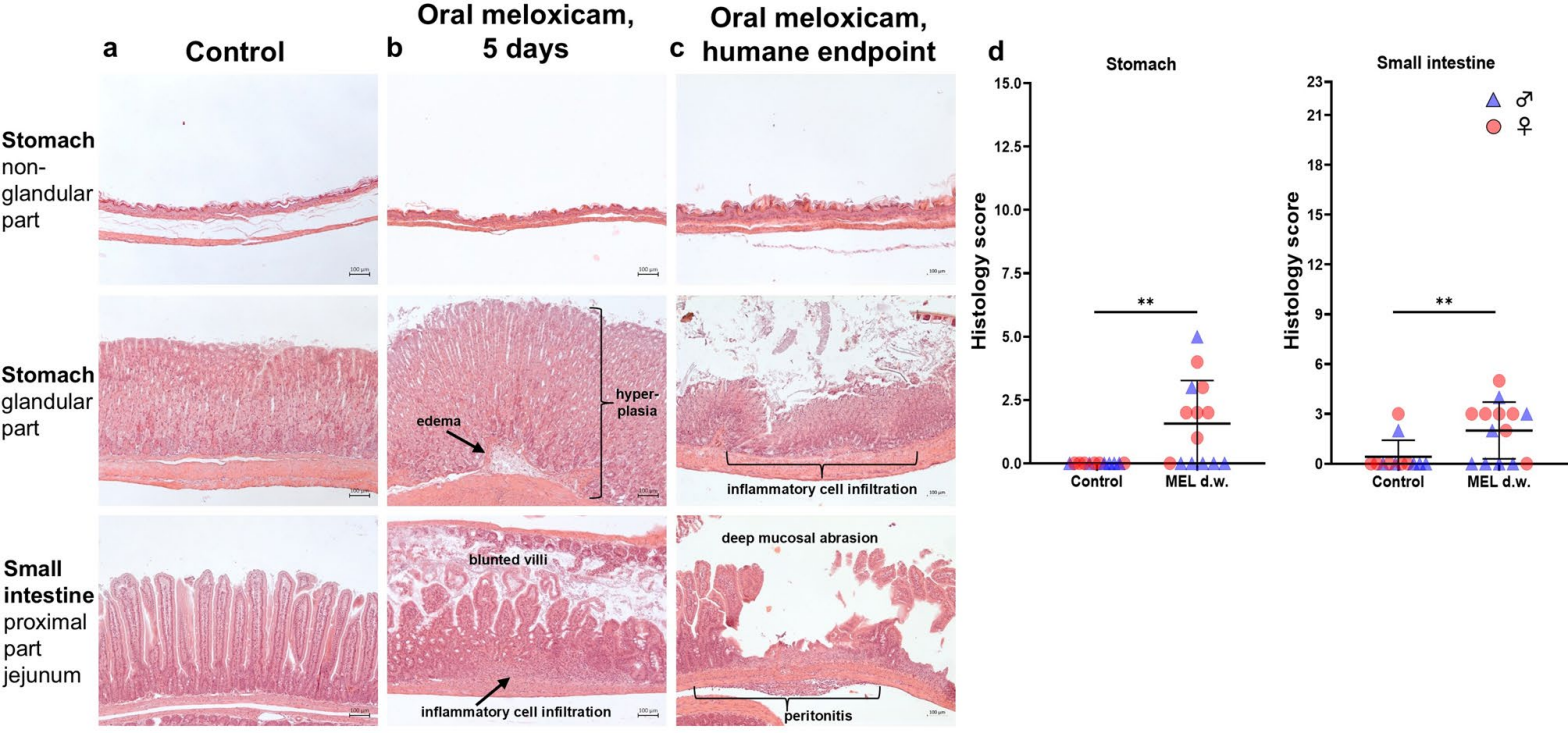
Prolonged oral meloxicam (MEL) treatment (20 mg/kg/24 h) has adverse effects on histology of stomach and small intestine. Representative hematoxylin–eosin staining of forestomach (upper panel), glandular stomach (middle panel), and proximal part of jejunum (bottom panel) of (**a**) control animals (left), (**b**) animals after 5 days of MEL (20 mg/kg/24 h) drinking water (d.w.) treatment (middle) and (**c**) one individual reaching humane endpoint at 96 h after start of treatment (right). Images of the control group have been used before in a previously published study^10^. (**d**) Histology score values of stomach and small intestine of male and female mice are significantly increased after 5 consecutive days of MEL d.w. treatment using one sample Wilcoxon test compared to untreated control group (control n = 6 per sex, MEL n = 7 per sex; **p < 0.01).

To assess potential side effects of continuous MEL treatment for 5 days via d.w. on organs of interest, the stomach, duodenum, proximal part of jejunum, liver, and kidneys of 7 randomly selected mice per sex were examined in detail by a veterinarian with pathological expertise. Representative images of stomach and jejunum sections from control and MEL-treated mice are shown in Fig. 6a, b. The stomach of treated animals showed mild to moderate infiltration of polymorphic cells restricted to the Lamina mucosa, hyperplasia of the Lamina mucosa and mild edema in the Lamina submucosa. The infiltrations occurred in a scattered pattern where normal areas of tissue were interrupted by the descripted changes. The small intestine of MEL-treated animals showed a mild to moderate infiltration of polymorphic cells mostly restricted to the Lamina mucosa and Lamina submucosa, again in a scattered pattern. Also, focal erosions with destruction of the epithelial lining of the mucosa were found in some animals. Images of one female mouse that had reached the humane endpoint at 96 h after treatment start are shown in Fig. 6c. In both animals that had to be euthanized as they reached humane endpoint, the histological analysis showed severe lesions in the descripted scattered pattern. Normal intestine tissue was interrupted by deep abrasions of the mucosa and marked appearance of polymorphic cells infiltrating all layers of the intestinal wall, causing a peritonitis. Statistical analysis revealed increased summation score of the stomach (n = 14; 1.57 ± 1.70; *p* = 0.0078) and the small intestine (includes duodenum and proximal part of jejunum, n = 14; 2.0 ± 1.71; *p* = 0.0039) compared to controls (stomach, n = 12; 0 ± 0; small intestine; 0.42 ± 1.0) (Fig. 6d). Histological examination of H & E stained-liver and kidney sections did not indicate adverse effects on these organs.

During autopsy of all remaining mice (21 males, 19 females) at the end of the d.w. treatment over 5 days, all organs were examined macroscopically. Here, 7/19 female MEL-treated mice exhibited apparently enlarged spleens. Further, 2/21 males and 1/19 females had a noticeable small stomach. In 5/21 males and 2/19 females the vessels of the gastrointestinal tract appeared strongly visible and 2/19 females had a soft and fragile tissue structure of the jejunum. As subsidiary finding, 1/19 females had a kidney cyst, which can be considered as normal background pathological finding in C57Bl/6J mice. In control mice, 1/6 females had a noticeable small stomach.

### Changes in red blood cell parameters, electrolytes and ALT but not corticosterone levels after prolonged oral self-intake of MEL.

A standard blood profile analysis was performed following final cardiac puncture at 120 h of MEL d.w. treatment and compared to controls. Blood profile values after five-day oral treatment are provided in Table 2. Comparison revealed lower RBC (10^3^/mm^3^) (control vs. MEL, *p* = 0.0255), HGB (g/dl) (control vs. MEL, *p* = 0.0007), HCT (%) (control vs. MEL, *p* = 0.0027), MCV (*f*m^3^) (control vs. MEL, *p* = 0.0356) and MCH (pg) (control vs. MEL, *p* = 0.0029) values in MEL-treated mice. Measurement in a second system confirmed reduced HGB levels and HCT in MEL-treated mice (Table 2). Electrolyte and metabolite data showed increased Ca^2+^ (control vs MEL, *p* = 0.0355) and Cl^−^ concentrations (control vs. MEL, *p* = 0.0004) in MEL-treated mice. Determination of the liver enzymes aspartate transaminase (AST) and alanine transaminase (ALT) revealed a significant decrease of ALT in the MEL-treated mice compared to controls (*p* = 0.0123). Corticosterone analysis did not show differences between both groups.

**Table 2:** Blood count, electrolyte, metabolite, enzyme and corticosterone analysis of naïve control mice (n = 6 male, 6 female) and meloxicam (MEL)-treated mice (n = 21 male, 21 female) performed after final cardiac puncture following a five-day oral MEL treatment. For details on the experimental design, see Fig. 1.

Parameter	Control	MEL	Analyzer
WBC (10^3^/mm^3^)	8.52 ± 3.41	7.57 ± 4.30	a
LYM (10^3^/mm^3^)	6.49 ± 2.38	5.51 ± 3.13	a
MO (10^3^/mm^3^)	0.38 ± 0.22	0.40 ± 0.25	a
GRA (10^3^/mm^3^)	1.65 ± 0.90	1.66 ± 1.04	a
RBC (10^6^/mm^3^)	10.99 ± 1.28	9.06 ± 2.79 *	a
HGB (g/dl)	15.16 ± 1.72	11.44 ± 3.42 ***	a
	15.86 ± 0.81	12.33 ± 1.74 ****	b
HCT (%)	55.82 ± 6.07	43.56 ± 12.93 ***	a
	48.81 ± 1.48	37.84 ± 5.34 ****	b
PLT (10^3^/mm^3^)	862.50 ± 440.70	958.2 ± 588.40	a
MCV (*f*m^3^)	50.75 ± 2.53	49.15 ± 2.17 *	a
MCH (pg)	13.83 ± 0.78	12.72 ± 1.14 **	a
MCHC (g/dl)	27.17 ± 0.44	25.87 ± 2.29	a
RDW (%)	14.14 ± 0.45	14.51 ± 1.02	a
MPV (*f*m^3^)	5.77 ± 0.14	5.78 ± 0.30	a
cNa^+^ (mmol/l)	152.60 ± 3.00	151.90 ± 6.39	b
cCa^2+^ (mmol/l)	1.58 ± 0.03	1.64 ± 0.07 *	b
cCl^-^ (mmol/l)	116.80 ± 2.59	121.20 ± 2.90 ****	b
cGlu (mmol/l)	12.64 ± 1.79	12.91 ± 6.64	b
cLac (mmol/l)	7.16 ± 2.05	6.37 ± 1.90	b
AST (U/L)	97.79 ± 80.87	80.41 ± 48.27	c
ALT (U/L)	23.71 ± 16.27	9.91 ± 5.26*	c
Corticosterone (ng/mL)	75.23 ± 49.15	63.94 ± 66.09	

WBC, white blood cells; RBC, red blood cells; HGB, hemoglobin; HCT, hematocrit; PLT, platelets; MCV, mean corpuscular volume; MCH, mean corpuscular hemoglobin; MCHC, mean corpuscular hemoglobin concentration; AST, aspartate transaminase; ALT, alanine transaminase. Values presented as mean ± SD; * *p* < 0.05, ** *p* < 0.01, *** *p* < 0.001, **** *p* < 0.0001. Data were tested for normal distribution using D’Agostino & Pearson test, Anderson–Darling test, Shapiro–Wilk test, and Kolmogorov–Smirnov test. Unpaired two-tailed Student’s t-test or Mann–Whitney test was used to test for differences between control and MEL-treated mice). a, scil Vet abc, scil animal care company GmbH, Germany; b, ABL815 Flex blood gas analyzer, Radiometer, Denmark; c, Cobas c111, Roche Diagnostics, Vienna, Austria. Blood count, electrolyte and metabolite analysis: control mice: n = 6 male, 6 female; MEL-treated mice: n = 21 male, 19/21 female (no values for two female mice, one due to device error and one reached humane endpoint where no blood sampling was performed). Enzyme analysis: control mice: n = 4 male, 3 female; MEL-treated mice: n = 5 male, 6 female. Corticosterone analysis: control mice: n = 5 male, 3 female; MEL-treated mice: n = 18 male, 10 female.

Significant values are in [bold]

## Discussion

In this study, PK and side effect profiles were determined in healthy mice of both sexes after a single s.c. injection of MEL. During prolonged administration of MEL via d.w., oral acceptance and effects on behaviors in the home cage were also investigated. After s.c. injection of 5 mg/kg, a short t_1/2_ below 2.5 h was found, with plasma levels remaining above or within an estimated therapeutic range for up to 6 h. The oral target dose intake of 20 mg/kg MEL per 24 h from sweetened d.w. was achieved, but plasma concentrations fluctuated widely over the 5 treatment days. Unwanted effects reflected by various changes in physiological, behavioral, blood and histological parameters were observed, particularly for the 5 days of oral treatment.

As both an approved species-specific drug formulation and a clear evidence for appropriate dosing of MEL in mice are lacking, we decided to examine currently recommended maximum doses^4,5^ for both administration routes. For s.c. injection of 5 mg/kg MEL, we here determined a t_1/2_ of 2.32 h for adult male and female C57BL/6 J mice, which is similar to that reported by others for lower MEL doses in juvenile mice, namely an average t_1/2_ of 2.22 h in 4–5 week old C57BL/6 mice of both sexes for 1.6 mg/kg MEL^7^ and a t_1/2_ of 3.08 h in adult female mice of the CD1 strain for 1 mg/kg MEL^13^. The observed C_max_ here is more than twice as high than previously reported for male Swiss Webster mice post single s.c. injection of 5 mg/kg MEL (C_max_ of 2519 ng/mL)^8^. This can be explained by the fact that the T_max_ found in the present study was already at 1 h while measuring plasma levels in Swiss Webster mice started only at 2 h after injection and therefore likely missed the peak concentrations^8^. In line with the observation of Chen et al.^7^, we found lower plasma concentrations in male mice early after injection, supporting the importance to include both sexes into PK studies. Even with the comparatively high dose applied here the short half-life leads to a period of only up to 6 h with plasma levels within or above an assumed therapeutic range (see below). This suggests, that other than currently practiced^3,16^, shorter than 12–24 h injection intervals would be necessary to maintain plasma levels within the therapeutic range for longer than just a few hours. A previous report applying a lower MEL dose came to a similar conclusion^7^. However, being hesitative with repeated MEL injections seems advisable in view of the risk for side effect induction as seen in the present study for prolonged treatment.

Non-invasive administration of analgesics via the d.w. represents a strategy to reduce stress and disturbance associated with parenteral administration routes. To our knowledge, this study is the first to report resulting plasma levels for successful voluntary self-administration of MEL in mice via this route. Indeed, more than 10 years ago, Ingrao et al.^12^ already explored this administration method in male C57BL/6 J mice for the same target dose and similar formulation (20 mg/kg/day, Metacam Injectable 5 mg/mL, Boehringer Ingelheim) but a shorter treatment period (36 h). However, the mice did not consume the MEL-containing d.w., indicating dislike of the taste. Therefore, we here sweetened the d.w., which overcame this limitation and achieved intake of the full target dose in male mice whereas female mice remained slightly below the intended intake. This observation is in line with recently reported voluntarily consumption of a flavored MEL-containing gel in mice^15^, which found acceptance of an average daily dose of 2.4 mg/kg (used formulation: Apex Meloxicam Oral Suspension for Dogs, 1.5 mg/mL, Dechra Australia) but did neither determine PK nor the circadian rhythm of gel intake. However, as acceptance alone does not inform about achieved plasma concentrations, we performed respective measurements repetitively throughout the 5 day-treatment, including sampling at the end of light and dark phases. Other than in our previous study on the NSAID carprofen^10^, the resulting plasma concentrations for MEL turned out to fluctuate greatly, showing high values at end of dark phases (24 h, 120 h) and low values at other time points. This was probably the consequence of both the circadian rhythm of water intake and the short elimination half-life of MEL, suggesting that oral self-intake is not a purposeful route of administration for MEL in C57BL/6 J mice.

Species-specific therapeutic plasma levels of MEL in mice corresponding to a robust analgesic effect are still unknown to date. Current literature often refers to an estimated range of therapeutic efficacy between 390 and 911 ng/mL derived from dogs and cats (IC_50_ 390 ng/mL, analgesic effect in paw inflammation model dog^23^; IC_50_ 911 ± 189 ng/mL, lameness score cat^24^). We administered relatively high MEL doses, and plasma levels were above or within this estimated range for up to 6 h after s.c. injection as well as in the mornings during oral self-intake. However, plasma level might not be considered the only reference for therapeutic efficacy, but also analgesic concentration in the tissue and enzyme inhibition, both of which were not determined here. Conflicting observations regarding the impact of MEL on post-operative pain in mice have been reported questioning its efficacy even at high doses. Dose recommendations for s.c. injections of MEL in mice range between 1 and 20 mg/kg with an injection interval of 6–12 h^5,6^. A study which applied single s.c. injection of 2 mg/kg indicates there might be sufficient analgesic coverage after immunization with Freund adjuvant according to analysis of behavioral parameters^28^. After craniotomy, a reduced mouse grimace scale up to 8 h after surgery suggests analgesic efficacy of 2–5 mg/kg s.c. injected MEL^29^. Following vasectomy, 20 mg/kg MEL administered s.c. 30 min prior surgery, successfully reduced mouse grimace scale and a pain score^30^, and corticosterone levels were comparable to control mice without surgery^31^. In contrast, doses of 5 and 10 mg/kg did not provide proper pain relief after vasectomy^31^. Accordingly, a study found that after laparotomy, doses of 1, 5, and 20 mg/kg as single s.c. injection were not effective to ensure appropriate analgesia^9^. Further studies investigating the clinical efficacy of MEL would therefore be necessary. In view of the side effect profile found here, it seems advisable to focus these studies on short-term treatment with MEL.

Not entirely unexpected, we observed side effects attributed to MEL treatment, particularly during prolonged administration via d.w.. Mainly female mice showed increased clinical score values reflecting impaired condition, including two individuals reaching humane endpoint before the end of experiment. The Irwin test revealed mild to moderate excitation, but also impaired coordination, and slight sedation, which was accompanied by intermittent reduction in wheel running activity, pointing to activity- and/or locomotion-reducing properties of MEL in mice. This is in agreement with observations made in an open field test, during which MEL-treated healthy mice (20 mg/kg s.c.) demonstrated reduction in exploratory activity^32^. We further observed slightly decelerated grooming activity after 72 h under oral MEL treatment. Overall, these findings indicate that prolonged MEL treatment reduces activity and might impact motor performance in mice. If these parameters are to be used to assess post-operative pain, it should therefore be taken into account that it will be difficult to differentiate between the effects of MEL and the effects of pain on them. The decrease in body weight was most likely mitigated by s.c. fluid injections performed in mice with increased score values. It can be assumed that without this, the decrease would have been more pronounced. As we did not use camera-based monitoring, i.e. photographs or videos, for MGS assessment we cannot exclude that MEL still effects the MGS since it has been shown that life scoring is less sensitive than remote assessment^33^.

Moreover, histopathological examination after 5 days of treatment revealed adverse effects on gastrointestinal tissue, such as inflammatory cell infiltration in the stomach and hyperplasia in the small intestine, pointing out that tolerability of prolonged MEL treatment at the investigated dose in mice is poor. Complementary, recently published data suggest reduced tolerability and severe side effects of MEL use in mice, such as a high risk to develop gastric ulcerations after repeated s.c. injections of 20 mg/kg for 3 or 7 days^18^. Also, during chronic administration of low-dose MEL (1 mg/kg daily) over 28 days via oral gavage, histopathological stomach and duodenal alterations as well as changes of cardiac and hepatic tissue, and genotoxic effects were described^19^. Another study reported that an initial dose of 20 mg/kg followed by repeated injections of 10 mg/kg caused acute mortality^14^. In contrast, s.c. dose injection of up to 20 mg/kg was described to be safe for application, but mice also developed concentration-dependent skin pathology at the injection site^17,32^.

Administration of MEL via d.w. also caused an increase in rectal body temperature of approximately 0.8 °C in male and 1.0 °C in female mice (male: 37.6 ± 0.9, BL2 vs. 38.4 ± 0.7 °C, MEL; female: 37.4 ± 0.8, BL2 vs. 38.4 ± 0.7 °C, MEL). We previously made a similar observation during treatment with the NSAID carprofen in mice with a body temperature rise in the same extent (around 1.0 °C)^10^. Although the observed change in body temperature did not appear to affect the animals’ wellbeing, it could serve as a potential confounder when using body temperature as evaluation parameter such as for post-operative pain or other distress. For example, a 0.5 °C increase in body temperature has been reported 12–24 h after laparotomy in mice without analgesia^34^.

Studies about MEL effects on hematologic parameters in mice are rare. Here, Ca^2+^ and Cl^-^ concentrations were slightly elevated, while in another study chronic oral MEL treatment for up to 7 days was not associated with serum chemistry changes^18^. Also, blood count after 5 days of oral treatment revealed reduced red blood cell numbers compared with control mice. Moreover, hemoglobin and hematocrit values were decreased, which might be associated with repeated blood sampling during our experiments^35^. In humans, prolonged treatment with some NSAIDs is associated with anemia due to gastrointestinal bleeding^36^ or induced hemolytic anemia^37^. Additionally, MEL at a dose of 20 mg/kg is potentially associated with a risk of gastric ulceration in mice^6^. On the other hand the histopathological examinations in the present study did not reveal signs of bleeding. Overall, the changes were significant but values still remained within reported reference ranges^38,39^. Moreover, the liver enzyme ALT values were significantly reduced compared to control mice. This might be in line with published studies suggesting that MEL treatment in diseased or compromised mice can indeed be associated with a reduction in ALT blood levels^40,41^. It is worth noting that control mice were housed under the same conditions, including continuous access to running wheels, but were not subjected to repeated blood sampling, tolerability assessments, or behavioral testing. Thus, we cannot exclude the possibility that the outcomes of blood parameter measurements would have been different, which may have affected the comparability with the experimental mice.

Plasma corticosterone levels are known to increase as part of the physiological response to stress^22^. Interestingly, despite the changes in clinical and physiological parameters, the prolonged MEL treatment was not associated with altered corticosterone plasma levels. This could mean that the stress induced by the observed side effects was either too low in severity, too short in duration or not the kind of stress that triggers a response in the form of altered corticosterone blood levels. On the other hand, we measured only at one time point, so that earlier changes might have been overseen.

Our study has some limitations. These include investigation of only one mouse strain and one dose of MEL. The strain C57BL/6 J was chosen as it is the most frequently applied strain in preclinical research^42^, aiming for our findings to be broadly applicable. However, as strain differences may exist regarding PK, oral acceptance, and tolerability of MEL, the present findings might not be fully translatable to other mouse strains. Although being of interest, the inclusion of several doses was beyond the scope of the present study. The highest doses recommended in the literature were selected as it seems to be most important to confirm or refute the tolerability of these high doses, as these are being used in preclinical research and carry the highest risk of side effects. Another limitation might be that we did not perform histological assessment after single s.c. injection. This is due to the longitudinal study design applied. In line with the 3Rs principles, this reduces the number of animals needed but may also decrease data variability relative to the use of separate experimental groups as each mouse and/or cage unit served as its own control. However, this approach also comes with a limitation. Comparing home-cage based behavior to baseline recordings instead of side-by-side control group carries the risk of being influenced by factors that change over time. Therefore, despite our efforts to minimize the impact of environmental factors, e.g., by providing four weeks of acclimatization and habituation during which the mice were exposed to running wheels, nesting material, and burrowing tubes, and all handling procedures, we cannot fully rule out that changes in home-cage based behavioral parameters, such as the reduced latency to start burrowing activity on day 5, were unrelated to MEL treatment.

Another drawback might be that we did not record all data on an animal-individual level. This is because we wanted to avoid single housing for animal welfare reasons. The decision to investigate on cage level for several parameters was resulting from outbalancing the importance of animal welfare (group housing) with the relevance to generate animal-individual data for all parameters. Since our investigations were limited to healthy animals, we cannot rule out the possibility that side effects in mice may be even more pronounced after surgery. Another limitation of this study is that the potential analgesic effect of the applied MEL dosages was not assessed in a (surgical) pain model. Such studies might be conducted in the future with caution and awareness of possible adverse effects, with the focus on short-term rather than long-term treatment with MEL.

In conclusion, PK data suggest plasma levels in a potential therapeutic range for up to 6 h after single s.c. injection of 5 mg/g MEL accompanied by good tolerability. Therefore, MEL may be useful for short-term treatment. In contrast, self-intake from the d.w. for 5 days led to instable plasma concentrations in consequence of circadian water intake and fast MEL elimination, meaning that this administration route is not suitable. Prolonged treatment was going along with limited tolerability due to sedative-like effects, impaired clinical condition, and inflammatory changes in the gastrointestinal tissues, particularly in female mice. Limited tolerability already in healthy mice questions MEL’s applicability in mice compromised after surgical intervention. In view of the present findings and of increasing reports about MEL-associated side effects, its usage for pain management should be avoided in mice at high doses, for repeated injections, or for oral administration. The use of analgesics with more promising PK and tolerability profiles, such as carprofen, appears to be advisable for (refining) analgesic therapy in this animal species.

## Methods

Majority of used materials and methods are equivalent to a previously performed study^10^. This implies that parts of described materials and methods might be identical. The age-matched untreated control animals used for the blood parameter and histological analyses were the same as used for the previous study.

### Animals, health monitoring and cage set-up

This study was approved by the responsible state authority (Niedersächsisches Landesamt für Verbraucherschutz und Lebensmittelsicherheit) under reference number 33.8–42502-04–21/3640. All methods were performed in accordance with the directive 2010/63/EU of the European parliament and of the council on the protection of animals used for scientific purposes and are reported according to the ARRIVE 2.0 guidelines (Animal Research: Reporting In Vivo Experiments; Essential 10). Breeding and housing conditions were carried out as previously described^10^. C57BL/6 J mice were purchased from the Institute for Laboratory Animal Science and Central Animal Facility, Hannover Medical School, Hannover, Germany, and bred in-house in an IVC system. After transfer to the experimental room, mice were housed in experimental groups of three female or male individuals per cage (type 3, macrolone, UNO BV, The Netherlands) in an open (conventional) cage system on standard bedding material (wood chips from spruce, poplar and aspen trunks, LAB.BED, Thomsen Räucherspäne Räucherholz GmbH & Co. KG, Germany) in a temperature-controlled facility (average temperature 20.8 °C, average humidity 48.0%) under a 14 h/10 h light/dark cycle (lights on: 6:30 a.m./lights off: 8:30 p.m.) with food (altromin 1320 standard diet, Altromin Spezialfutter, Germany) and filtered (particle filter, 5 µm pore size) tap water (non-acidified) ad libitum.^10^ To minimize potential maintaining-associated confounders, cages were changed weekly, and home cages were always returned to their original position on the cage shelf. The home cages were all placed on the same side of the animal room and were positioned in such a way that comparable lighting conditions were ensured. As sex-based differences in PK and behavior have been reported, both sexes were included in our study^20,43,44^. Routine health monitoring according to FELASA recommendations^45^ did not reveal any evidence of infection with common murine pathogens except for murine astrovirus, Pneumocystis murina, Rodentibacter sp., and apathogenic intestinal flagellates. For routine health monitoring, immunocompetent sentinel animals are used. These “bedding sentinels” receive 100% dirty bedding (i.e. used cages including nest, food and drinking bottles), and per quarter at least one of the sentinels is tested and replaced. The mouse strain used as sentinel is changed every quarter to provide a broad spectrum of different pathogen susceptibilities. In addition to the bedding sentinels, immunodeficient colony animals are used on an annually basis for bacteriology and PCR analysis for mouse hepatitis virus, murine norovirus, parvoviruses, and Theiler´s murine encephalomyelitis virus. Furthermore, AED (exhaust air dust) material is tested for pathogens according to FELASA recommendations including rodent-specific Helicobacter spp. by qPCR analysis. Moreover, animals suffering from clinical symptoms undergo full necropsy procedures including gross pathology as well as testing for infectious agents. Experimental group size included 21 male and 21 female mice. Due to reaching humane endpoint of two female mice at 48 and 96 h after start of d.w. treatment, number of animals per cage was reduced to 2 individuals for respective cages from that time point. As control for histologic analysis and blood profiles, 6 male and 6 female age-matched C57BL/6 J mice from a previous study^10^ kept under the same housing conditions, including running wheels, were used. These animals were not subjected to repeated blood sampling, tolerability or behavioral tests. No targeted randomization method was used to allocate the mice to experimental and control groups in the breeding area. The mice were randomly allocated to groups of 3 by the animal keepers in the breeding area after weaning from the dam. Mice were transferred from the breeding area to the experimental room 7 weeks before start of the experiments. This time was subdivided into four weeks of acclimatization to the new room and housing conditions without particular handling, followed by three weeks of habituation (adaptation) to all handling procedures (Fig. 1). Each mouse was adapted three times to weighing, manual fixation and restrainer fixation. In their home cages, the mice had continuous access to nesting material, running disks installed on plastic houses, and wooden gnawing material as additional enrichment (plexx B.V. Aspen Bricks S, the Netherlands). Cage and running wheel set up was as previously described^10^. At start of experiments with age of 13 weeks, mice weighed 27.7 ± 1.2 g (male) and 21.7 ± 0.8 g (female).

### General experimental design and blood sampling procedure

An overview of the experimental setup is provided in Fig. 1. The general experimental design followed previously published methods^10^. Animals were habituated to all handling procedures and interventions and were trained for burrowing before experiments started. Running disks were accessible constantly from beginning of habituation. In the longitudinal study design, the individual baseline data from each mouse or cage unit, respectively, were used as its own control. During baseline phase 1 (BL1), data of all parameters of interest were obtained over 5 days, including food and water consumption, body weight, nest score, Irwin test, mouse grimace scale, body temperature, wheel running and burrowing activity. This was followed by s.c. injection and subsequent blood collection for PK analysis at 1, 2, 3, 6, 12, 24, and 48 h post injection. After 2 weeks of recovery, a second baseline phase (BL2) was performed identical to BL1. In weeks 3 and 4 (recovery phase), each mouse was monitored and handled twice per week, including body weight measurement, clinical scoring, and renewal of identification by pen marking on the tails. Detailed habituation was not resumed during this phase. Cage equipment remained as before. Then, MEL was administered via d.w. for 5 days, and blood was collected at 3, 6, 12, 24, 36, 108, and 120 h after start of administration. Blood was sampled from a lateral tail vein by scalpel micro-incision, which has been shown to be of lower burden to the animals than withdrawal from the facial vein or the retro-orbital sinus^46^. Blood was collected in EDTA tubes (Sarstedt 200 µL Microvette®, Germany) at all sampling time points. At each collection time point (see above), samples (~ 120 µL) were taken from 6 mice (3 male, 3 female). For sampling time point 108 h after start of d.w. treatment, blood was collected from only two female mice, as one reached humane endpoint at 96 h. Immediately after sampling, blood-containing tubes were placed on ice, then centrifuged (2500xG/10 min/4 °C) and plasma was stored at − 80 °C until further processing. Directly prior to blood sampling, tests for tolerability (Irwin test) were conducted in the respective 6 mice. At the end of the experiment, following the five-day oral MEL treatment, mice were killed by inhalation and subsequent cardiac puncture. Despite some concern about animal welfare using this euthanasia method, there is insufficient evidence for unbiased assessment of CO_2_ inhalation effects during euthanasia on welfare indicators in laboratory mice^47^. Still, euthanasia by CO_2_ inhalation is an accepted and commonly used methods, which is in accordance with Annex IV of the directive 2010/63/EU and conforms to the most recent “AVMA guidelines for the euthanasia of animals: 2020 edition”^48^. However, the method allows for the mice to stay in their home cage without additional handling stress. The flow rate used was low and adjusted to the volume of the home cage (3 L CO_2_ /min)^49^. Final blood samples were used for full blood count and analysis of electrolytes, glucose, and lactate. Further, necropsy of each animal including macroscopic investigation for abnormalities was performed. Stomach, small intestine, liver and kidneys were dissected for histological processing.

### MEL dosing and administration procedure

The MEL dosage both for s.c. and oral administration was chosen based on the current GV-SOLAS expert information on pain management for laboratory animals^4^ and current literature review^5^. Of the suggested dose ranges for mice (2–5 mg/kg s.c. or 10–20 mg/kg per os), the highest doses were applied. A commercially available MEL solution (Boehringer Ingelheim, Metacam® 2 mg/mL, injection solution for cats) was diluted with 0.9% sterile sodium chloride (B. Braun, Germany) to achieve a 10 mL/kg injection volume. The s.c. injection dose was 5 mg/kg. For d.w. administration, the injection solution (Boehringer Ingelheim, Metacam® 20 mg/mL, injection solution for cattle, horses and pigs) was diluted in filtered tap water (non-acidified), targeting a MEL dose of 20 mg/kg/24 h. MEL concentration calculation for group-housed mice (3 mice/cage, 7 cages per sex) was based on mean water consumption/24 h measured over 5 days (BL 2; male mice, 22.1 mL; female mice, 16.9 mL) and mean body weight (male mice, 29.4 g; female mice, 23.4 g). MEL-medicated d.w. was initially offered between 7:00 and 8:00 a.m. and provided continuously for 5 consecutive days. The medicated water was freshly prepared every morning in red polyphenylsulfon water bottles (Tecniplast, Italy). MEL-medicated water was sweetened with 0.01% saccharine (≥ 98%, Sigma-Aldrich®), as MEL-medicated water might be consumed in reduced amounts by mice^12^.

### Sample preparation and liquid chromatography mass spectrometry analysis

MEL content in plasma was quantified by liquid chromatography mass spectrometry (LC–MS/MS) basically as described previously for carprofen^10^. Plasma samples and analgesic calibrators (25 µL) were thawed and diluted using 100 µL extraction solvent (acetonitrile/ methanol 1/2) containing 6.25 nM moclobemide (obtained from Sigma-Aldrich Chemie GmbH, Taufkirchen, Germany) as internal standard (equals a final concentration of 10 nM/sample) in a 1.5 mL reaction tube (SafeSeal®, Sarstedt, Germany) for analyte extraction and protein denaturation. Samples were mixed for 30 s using a vortex mixer and frozen overnight at − 20 °C to complete protein precipitation. Then, samples were thawed and centrifuged for 10 min at 20,800 × g/4 °C for protein separation. For mass spectrometry analysis, samples were diluted 1:500 using dilution solvent (acetonitrile/methanol/water 2/2/1) containing 5 nM internal standard, and 100 µL were transferred into mass spectrometry vials (Wicom, Heppenheim, Germany) with inserts (Macherey–Nagel, Düren, Germany) for MEL quantification. This involved chromatographic separation on a reversed phase C18-column (ZORBAX Eclipse XDB-C18 1.8 µ, 50 × 4.6 mm, Agilent, Santa Clara, California, USA) connected to a C18 security guard (Phenomenex, Aschaffenburg, Germany) which was kept on 40 °C during the whole analysis. A linear gradient was performed using an HPLC-system consisting of two LC-30AD HPLC pumps, a SIL-30AC temperature controlled autosampler, a DGU-20A5 degasser, a CTO-20AC oven, and a CBM-20A control unit (Shimadzu, Duisburg, Germany). 10 µL of the sample was injected. Elution started with 80% of solvent A (water + 0.1% formic acid). Within 7 min, the amount of solvent B (methanol 0.1% formic acid) was linearly increased to 95%. This composition was maintained for 3 min followed by a 3 min re-equilibration of the column back to 80/20 (solvent A/solvent B). The total analysis time was 13 min at a flow rate of 0.4 mL/min. The retention time of MEL was 6.84 min and of moclobemide (internal standard) was 3.54 min. Analytes were detected by triple quad mass spectrometry (5500QTRAP; Sciex, Framingham, Massachusetts) in multiple reaction monitoring (MRM) mode. Ionization was achieved using positive electrospray ionization at 400 °C. For MEL, the mass transition m/z 352 → 115 was optimized for quantification, and for moclobemide, the mass transition m/z 269 → 182 was used. Control of HPLC and the mass spectrometer as well as data sampling was performed by Analyst software (version 1.7., Sciex). For quantification, calibration curves were created by plotting peak area ratios of MEL, and the internal standard versus the nominal concentration of seven calibrators containing 0.245–503 nM MEL (corresponding to a lower limit of quantification of 0.09 ng/mL and an upper limit of quantification of 177 of ng/mL) prepared in mouse plasma. The calibration curve was calculated using quadratic regression and 1/x weighing.

PK analysis was performed using the PKanalix application (Version 2024R1, Lixoft, Antony, France). Noncompartmental analysis was applied for determination of plasma elimination half-life of MEL.

### Tolerability assessment and side effects

#### Irwin test battery, body temperature, and mouse grimace scale

Prior to blood sampling, tolerability of MEL was examined by a modified Irwin test battery^50^ at 1, 2, and 3 h after s.c. injection and at 3, 6, 12, 24, 36, 108, and 120 h after start of voluntary uptake via d.w. as previously described^10^. The Irwin test is a behavioral test battery widely used by pharmaceutical industry to determine whether subjects show adverse effects from candidate compound treatment. The test included observations without handling, followed by assessments in an open field, followed by handling-based assessment of parameters, as described in detail previously^10^. For analysis, parameters were assigned to four different categories, i.e. excitation, coordination, sedation, and autonomous system. Mouse grimace scale was scored as part of the Irwin test assessment, and visually assessed (without handling of the mice) in the home cage according to established mouse grimace scale score^51^. Immediately after the Irwin test procedure, rectal body temperature was measured (PhysioSuite® for mice and rats, Kent Scientific Corporation, USA). For visualization of Irwin test, a heat map was created showing percentage of changes per investigated time point after treatment (n = 3 male and 3 female per time point, except d.w. treatment at 108 h (3 male, 2 female)). For further analysis of the Irwin test outcome, a sum score of each individual mouse and test category (excitation, coordination and sedation) was generated. Total score values were added for a total sum score. Negative score values were handled as positive score values for addition to sum score. Test parameters for which presence or absence were recorded were given 2 points if they deviated from normal. Handling associated vocalization was compared to animal individual BL and assessed with 2 points when deviant from BL. Vocalization during reflex testing (eyelid, pinna) was scored with 2 points when present. Presence of unusual behavior documented in free text (fasciculation) was scored with 2 points per observed behavior. One BL dataset was obtained and used for statistical analysis.

#### Food and water intake, and clinical scoring

Food and water consumption were gravimetrically measured every 24 h per cage during baseline phases (BL1 & BL2). During substance administration via d.w., water bottles were additionally weighed at blood sampling time points (for respective cages), resulting in fluid consumption data for day and night. Clinical score was determined twice a week during baseline (BL1 & BL2) phases and daily during MEL administration phases. Clinical scoring included judgement of activity, general state of health, behavior, body posture and body weight (Supplementary Fig. S2). As supportive measure, mice reaching clinical score 2 and above got s.c. fluid injections (0.5 mL Sterofundin®, Braun, Germany) and were monitored daily, individuals with score 3A were monitored twice per day. A score 3B and above was determined as humane endpoint leading to immediate euthanasia.

#### Home-cage behaviors other than grimace scale

A variety of behavioral parameters, such as burrowing, nesting, and grooming behavior, and wheel running activity have been suggested as potential indicators of pain in laboratory mice^20^. They were included in this study to determine potential impact of MEL treatment on their outcome in healthy mice. To avoid impact of cage change on behavioral parameters, cages were changed already 2–3 days prior to start of assessment (BL1 & BL2, MEL via d.w.). Nesting and gnawing material were provided continuously throughout the whole experiment.

#### Voluntary wheel running

Commercially available angled mouse running discs and igloos were purchased from ZOONLAB GmbH (Castrop-Rauxel, Germany). Running time (h:min:sec), running distance (km), average velocity (km/h) and maximum velocity (km/h) were recorded as previously described^52–54^ by bike computers (Sigma BC 16.16 STS, SIGMA-ELEKTRO GmbH, Germany). In brief, the sensor was fixed on the outside of the cage in close proximity (< 1 cm) to the magnet (neodymium, 10 × 3 mm), which was glued to the running disc. To avoid any accidental shifting of the igloos, the position of the setup in the cage was secured by a customized pedestal (polyethylene) which was attached to the cage floor with adhesive tape^10^. Wheel running parameters were continuously recorded and read out in the morning between 8:00 and 9:00 a.m. to obtain data during “dark” phase (lights on at 06:30 a.m.) and in the afternoon between 4:00 and 6:00 p.m. to record running behavior during “light” phase (lights off at 08:30 p.m.) over five consecutive days. Running time, running distance, and average running velocity was normalized to total time (h) during read out intervals (8 h during day and 16 h during night). Mice had continuous access to running discs during the complete experimental period. The first BL measurement in this study was performed 1 week after equipment of home cages with running disks. Data were recorded twice for baseline (BL1 & BL2) and under MEL treatment. Only the second baseline (BL2) recording was used as reference for data analysis.

#### Burrowing activity

Burrowing procedure was performed to investigate motivated goal-directed behavior^20^ during substance administration according to a modified protocol from Deacon et al.^55^. For assessment of burrowing behavior, empty transparent bottles (volume 330 mL, length 15 cm, diameter 5 cm, polycarbonate) were filled with food pellets (altromin 1320 standard diet, Altromin Spezialfutter, Lage, Germany). First, mice were habituated twice to burrowing overnight by placing food-pellet filled bottles in the front left corner of the cage. Next morning, bottle and food were removed. For experiments, bottles were filled with 151 ± 2 g (mean ± SD) food pellets, placed in the cage and removed after 2 h. Latency to start of burrowing (min) was visually measured for the first 30 min. In cases where the mice started burrowing later, 30 min were used as value for data analysis. At the end of burrowing procedure (after 2 h) the remaining pellets in the tube were weighed and the amount burrowed (%) calculated. Burrowing procedure was performed between 11:00 a.m. and 1:00 p.m. according to a previously described protocol^56^. Two baseline data sets (BL1 & BL2) were recorded for each cage, BL2 was used for statistical analysis. During oral substance administration, burrowing behavior was assessed every day for 2 h (at 24 h, 48 h, 72 h, and 96 h).

#### Nest building

Nesting behavior was observed to detect alterations of intrinsic motivated behavior under drug treatment according to an established nesting score^10,27^: 1 = no cotton pieces grouped together; 2 = cotton pieces paired together in one or two pairs; 3 = 3 cotton pieces grouped together; 4 = all cotton pieces grouped together; 5 = all four cotton pieces grouped together and completely shredded. Assessment of nest building test was performed using four cotton rolls (Ø 12 mm × 37 mm, ANT Tierhaltungsbedarf, Germany) per cage. Existing nesting material was taken out and four new cotton rolls were provided 10 h after start of MEL treatment via d.w. and nest score was recorded for the first time 2 h later. Subsequently, nests were scored every day in the morning (8:00–9:00 a.m.; 14, 38, 62, 86, 110 h after administration of nesting material) and in the afternoon (4:00–6:00 p.m.; 24, 48, 72, 96 h after administration of nesting material)^27^. Two individual baselines (BL1 & BL2) of nesting were performed, for statistical analysis median and range of BL2 was used. For illustration, median and range of nest scores are shown in Supplementary Fig. S4b up to 72 h after administration of nesting material, as nest scores did not change afterwards. In males, two trials with freshly provided nesting material were performed under MEL treatment (I and II), median and range of each trial is displayed. The second trial four new cotton rolls started 72 h after start of MEL d.w. treatment. For statistical analysis, both trials were individually compared with corresponding BL2 timepoints.

#### Grooming transfer test

Grooming behavior as potential indicator for pain or distress was evaluated according an established score^10,27^. Two baseline trials (BL1 & BL2) were performed. For statistical analysis, median and range of BL2 is displayed. At 10 h after start of MEL administration via d.w., 8 µL of fluorescent GloGerm Oil (Glo Germ Company Utah, USA) were applied onto the skin of to the neck region (first trial). When all mice had reached the highest possible score value (score 5), the fluorescent was applied again and a second trial was performed. Using an UV flashlight, (UVL 1006, Glo Germ Company Utah, USA), 2 h after application and then every day in the morning between 8:00 and 9:00 a.m. (14, 38, 62 h after application) fluorescent oil distribution was scored according to an established grooming score^27^ 1 = Fluorescence is strong at the application site on the forehead between the ears; 2 = Fluorescence is present at the application site and front, and/or rear nails; 3 = Fluorescence is at the application site and the ears, signal may be present at front and/or rear nails; 4 = Fluorescence is absent from the nails and ears but traceable amount remains at application site; 5 = Fluorescence is no longer visible. Two male mice with alopecia in the neck region were excluded from analysis.

### Final blood analysis

After CO_2_ induced asphyxiation, blood from final cardiac puncture was sampled in EDTA tubes (Sarstedt 500 µL Microvette®, Germany) and immediately stored on ice until analysis, to generate full blood count (scil Vet abc, scil animal care company GmbH, Germany) including leukogram of white blood cells (WBC), lymphocytes (LYM), monocytes (MO) and granulocytes (GRA). Red blood cell (RBC) count includes hemoglobin (HGB), hematocrit (HCT), platelets (PLT), mean corpuscular volume (MCV), mean corpuscular hemoglobin (MCH), mean corpuscular hemoglobin concentration (MCHC), red cell distribution width (RDW), and mean platelet volume (MPV). In parallel, additional blood analysis provided status of electrolytes (concentration of Na^+^, Ca^2+^, Cl^-^), metabolites (glucose and lactate concentration) and blood (hemoglobin, hematocrit) (ABL815 Flex blood gas analyzer, Radiometer, Denmark). Here, blood after cardiac puncture was sampled in capillaries (safeCLINITUBES, Radiometer, Denmark) and stored at room temperature (< 2 h) until analysis.

Additional blood from final cardiac puncture was sampled in EDTA tubes (Sarstedt 500 µL Microvette®, Germany), which were immediately placed on ice after sampling, then centrifuged (2500 × g/10 min/4 °C), and plasma stored at − 80 °C until further processing for aspartate transaminase (AST) and alanine transaminase (ALT) measurement. The samples were thawed, and the levels of AST and ALT were analyzed with a Cobas c111 analyzer using manufacturer systems 04,718,569,190/ALTL and 04,657,543,190/ASTL (Roche Diagnostics, Vienna, Austria) for ALT and AST, respectively. The lower detection limit for both AST and ALT was 2 U/L. As the required 170 µL of plasma were not available from all mice, AST and ALT determination was performed for 4 male and 3 female control mice as well as 5 male and 6 female MEL-treated mice.

### Corticosterone extraction and quantification

Corticosterone concentrations in plasma following diethylether extraction were analyzed with a corticosterone enzyme immunoassay (EIA) according to Palme and Möstl^57^, for all mice with enough remaining plasma (control mice: n = 5 male, 3 female; MEL-treated mice: n = 18 male, 10 female). All reagents were purchased at Sigma-Aldrich/Merck (Vienna, Austria). In brief, 1 mL of diethyl ether was added to 50 µL plasma and the resulting mixture vortexed for 20–30 s until two layers became miscible. The mixture was placed in a -20 °C freezer for 30–45 min, and the top layer containing extracted corticosterone was transferred to a new tube. Following evaporation at 60 °C the powdered corticosterone was reconstituted in 250 µL of assay buffer and stored at − 20 °C for future quantification. Aliquots of the extract were then analysed in the corticosterone EIA in duplicates. Samples were rerun when their CV exceeded 10% and the interassay CV of a pool samples was 9.4%. The sensitivity of the whole method was 0.1 ng/ml.

### Necropsy and histology

Directly after cardiac puncture, a routine dissection and visual inspection of the organs in the thoracic and abdominal cavity was performed for all mice. Thereafter, stomach, duodenum, jejunum, liver and kidneys were immediately removed and fixed in formaldehyde solution (4%). The small intestine was flushed with sodium chloride (0.9% Braun, Germany) and prepared in an improved swiss-roll technique^58^. After at least 3 days of formaldehyde fixation, tissues were trimmed according to the RITA-Guidelines^59–61^, dehydrated (Shandon Hypercenter, XP, Microm GmbH, Germany), and subsequently embedded in paraffin (Histoplast, Thermo Scientific, UK). Sections (2—3 μm thick, microtome Leica RM 2245, Germany) were deparaffinized in xylene and H&E stained according to standard protocols. In order to analyze a representative sample of 14 individuals, from each cage, organs of one mouse were randomly chosen for histopathological analysis, excluding the two mice that had reached humane endpoint (resulting in 7 male and 7 female mice assessed). A trained veterinarian with pathological expertise (SG) performed blinded evaluation (Axioskop 40, Carl Zeiss AG, Germany) and scoring of the sections (stomach, intestine). The kidneys and liver were evaluated for overall pathological findings. The scoring of the small intestine and stomach considered three general criteria according to a published protocol (cf. Table 1 of^62^): (1) inflammatory cells, (2) intestinal architecture, and (3) degree of ulceration, if present.

The criterion (1) inflammatory cells was subdivided into evaluation of severity and the maximal extent of the inflammatory cells regarding the histologic layers of the mucosa and each graded from 0 to 4, resulting in a maximum possible score of 8. The criterion (2) intestinal architecture was subdivided in evaluation of the epithelial and the mucosal layer and each graded from 0 to 4, resulting in a maximum possible score of 8. The criterion (3) degree of ulceration included score values from 0 to 3 and the area involving ulceration included score values from 0 to 4. Score values for all criteria per organ were added up, resulting in an overall maximum score of 15 for the stomach and 23 for the intestine. Representative images of histological sections were taken on an Axioskop 40 microscope utilizing the software ZEN (Version 3.5 Blue Edition, Carl Zeiss AG, Germany).

### Statistical analysis

Animal-individual data were recorded for MEL plasma levels, body weight, clinical score, grooming activity, Irwin test parameters, mouse grimace scale, body temperature, hematology, and histology, whereas data for food and fluid consumption, nesting, burrowing, and wheel running activity were recorded on cage level. For food and fluid consumption, data from two cages were two individual mice reached humane endpoint, were excluded from statistical analysis (72–120 h and 120 h). Results are presented as mean and data points (plasma concentration–time curves); mean ± standard deviation (MEL intake, wheel running, burrowing, body temperature, body weight, blood parameters, fluid and food consumption); median and individual data points (sum score Irwin test); or median and range (nest score, grooming score). To test for normal distribution of data, D’Agostino and Pearson test, Anderson–Darling test, Shapiro–Wilk test, and Kolmogorov–Smirnov test were conducted. Comparisons of two groups were performed by unpaired two-tailed Student’s t-test or Mann–Whitney test (blood parameters), or Wilcoxon matched-pairs signed rank test (body temperature). Multiple comparisons were analyzed by one-way ANOVA followed by Šídák’s multiple comparisons test (MEL plasma level sex difference), one-way Friedman-ANOVA followed by Dunn’s multiple comparisons test (burrowing, nesting, clinical score) or Dunnett’s multiple comparisons test (time to reach grooming score 5), or Kruskal–Wallis test followed by Dunn’s multiple comparisons test (sum score Irwin test). Further, multiple comparisons were analyzed by two-way ANOVA followed by Šídák’s multiple comparisons (wheel running, grooming activity, food, water).

GraphPad Prism 10 (Version 10.3.1, GraphPad Software, USA, GraphPad Prism) was used for all statistical analyses. A *p*-value < 0.05 was considered statistically significant.

During group allocation, experiment conduction, and data analysis, experimenters (AG, MB) were not blinded. Blinded experimenters performed analysis of MEL plasma concentration using LC–MS/MS (HB), ALT, AST (AK), and corticosterone measurement (JH, AP, RP), as well as histopathological evaluation (SG).

## Supplementary Information

Below is the link to the electronic supplementary material.

Supplementary Material 1

## Data Availability

All data files will be available from the RepoMed data repository of Hannover Medical School (10.26068/a79v-st68).
