# Supplementary material for: Suitability of prolonged meloxicam treatment in mice seems limited due to unfavorable pharmacokinetics, side effects, and impact on home-cage behaviors
Source: Sci Rep. 2025 Nov 7;15:39000. doi: 10.1038/s41598-025-25180-4 (PMC12594854; doi:10.1038/s41598-025-25180-4)
Supplement: Supplementary file 1 — Supplementary Material 1 [file 41598_2025_25180_MOESM1_ESM.pdf]

Supplementary information

Supplementary Figure S1

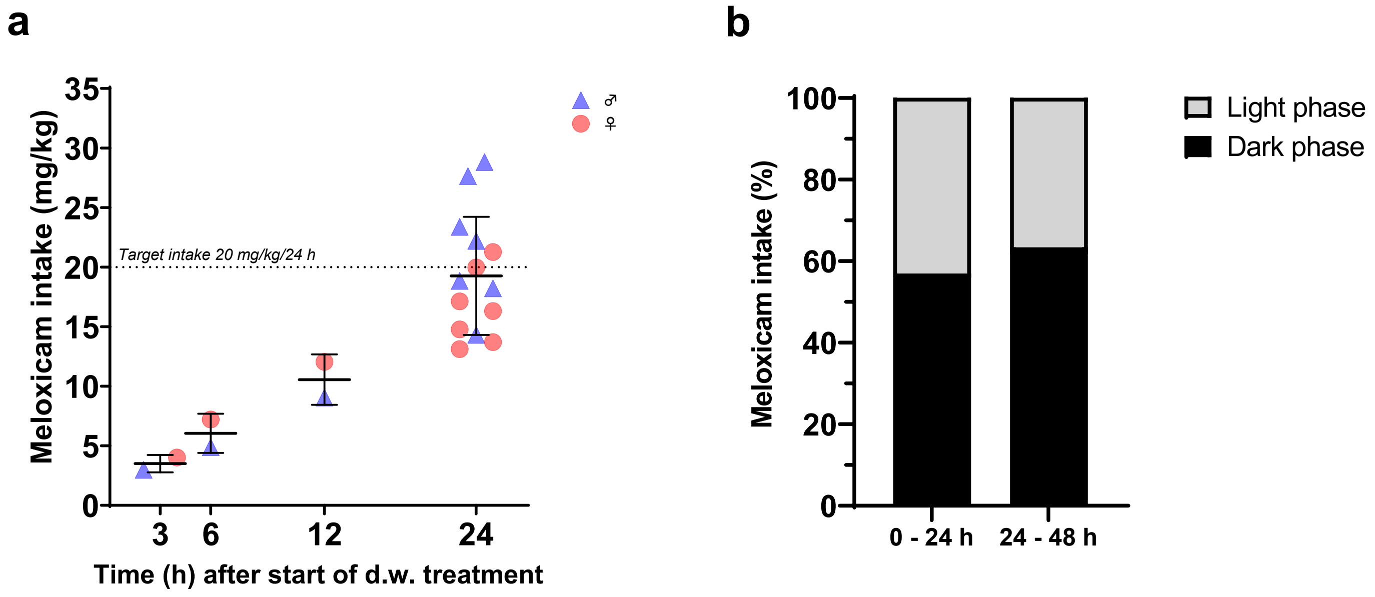

Supplementary Figure S1. Oral meloxicam intake from drinking water (d.w.) during light and dark phase. a Meloxicam intake (mg/kg) within the first 24 h during d.w. treatment. Dose was calculated from individual water intake and concentration per cage and sex. **b** Meloxicam intake (%) during the light and dark phase is displayed for 12 h intervals of total intake within 24 h (mean).

Supplementary Figure S2

	Clinical score	Activity	General condition	Behavior	Body posture	Body weight
	**1**	Very active	Clear eyes; clean orifice	Alert, curios, species-specific	Normal, species-specific movements and posture	Weight gain; No change; Weight loss ≤ 5 %
	**2**	Active				Weight loss ≤ 10 %
	**3A**	Slightly reduced activity	Eyes partly closed	Alert, hypo-/hyperlocomotion, slightly reduced grooming	Normal, slightly curved dorsal line	Weight loss ≤ 15 %
Humane endpoint	**3B**	Distinct reduced activity	Eyes partly closed	Frequent stops while movements, reduced food / water intake, reduced grooming	Slightly curved dorsal line	Weight loss ≤ 20 %
	**4**	Slow	Eyes partly closed; dirty orifice	Reduced reaction to stimuli, absence of grooming behavior, functional loss of extremities	Curved dorsal line	Weight loss ≥ 20 %
	**5**	Apathetic	Eyes closed; moist/clotted orifice	Self-isolation, no or negligible activity	Distinct curved dorsal line	Weight loss ≥ 20 %
	**6**	Moribund	Eyes closed; flat breathing	No activity, no reaction to stimuli	Lateral position, seizures, animal is cold	Weight loss ≥ 20 %

Supplementary Figure S2. Clinical score. Clinical score was assessed daily during subcutaneous and drinking water treatment. Reaching humane endpoint (dotted line), mice were killed immediately according to study approval by the responsible state authority.

Supplementary Figure S3

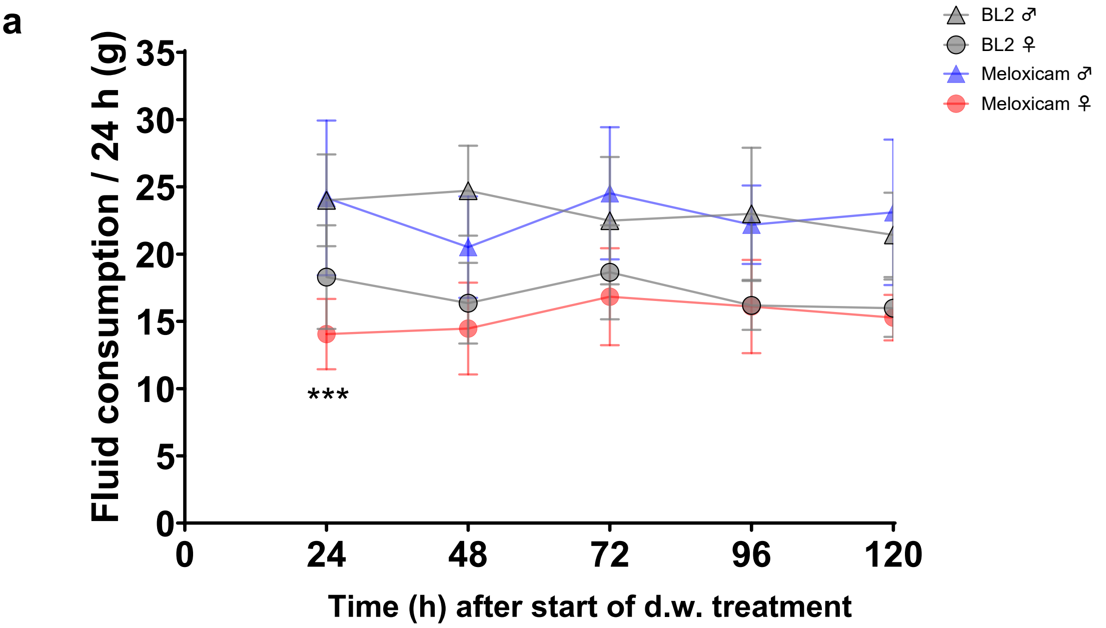

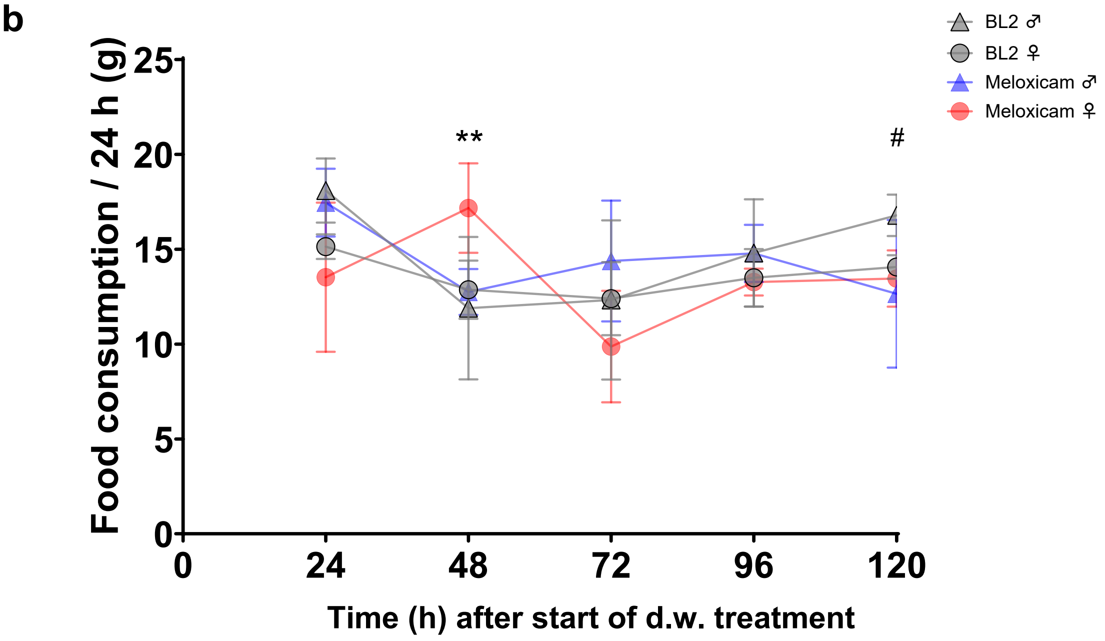

Supplementary Figure S43. Fluid and food consumption. a Fluid consumption (g) of drinking water/24 h during baseline 2 (BL2) and meloxicam-medicated water (target dose of 20 mg/kg/24 h) is presented over 5 consecutive days for male (♂) and female (♀) mice. **b** Food consumption/24 h (g) during BL2 and during meloxicam treatment is presented over 5 consecutive days for male (♂) and female (♀) mice. Data are shown as mean ± SD (n = 7 cages/sex; n = 3 mice/cage). Two-way ANOVA followed by Šídák's multiple comparisons test was performed to test for differences between BL2 and meloxicam treatment and reveals decreased fluid consumption at 24 h in females (*** p = 0.0010), increased food consumption at 48 h (** p = 0.0028) in females and decreased food consumption in males at 120 h (# p = 0.0133). Two cages with only two female mice were excluded from statistical analysis from time point 72 h until end of experiment or from time point 120 h, respectively

Supplementary Figure S4

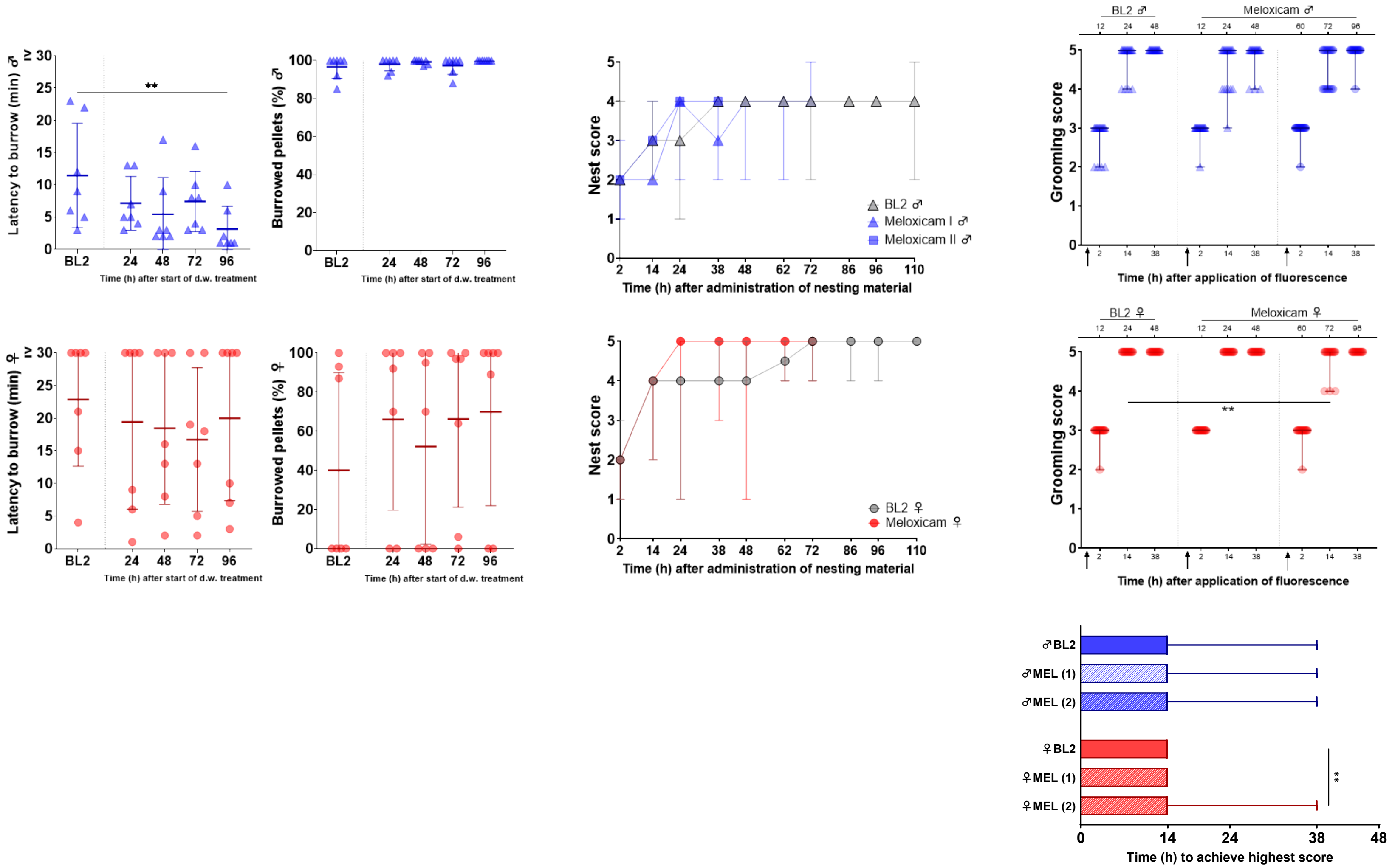

Supplementary Figure S4. Minor influence of oral meloxicam (MEL) treatment (20 mg/kg/24 h) via the drinking water (d.w.) on behavioral parameters. a Latency time (min) to start of burrowing behavior (left) after placing the burrowing tube in the cage and volume of burrowed food pellets (in % after 2 h) (right) is presented for male (upper graphs) and female (lower graphs) mice (n = 7 cages per sex, 3 mice/cage). Data are shown as mean±SD. Repeated measures Friedman ANOVA and Dunn`s multiple comparisons test does not detect statistical significant differences of burrowing performance compared to baseline 2 (BL2) except for shorter latency in males at 96 h after treatment start ($p = 0.0039$). **b** Nest score was not negatively influenced by oral MEL treatment in male (upper graph) and female (lower graph) mice (n = 42; 21 male, 21 female). Nesting material was provided 10 h after start of d.w. treatment. Data are presented as median and range (n = 7 cages per sex, 3 mice/cage). Data are shown as median and range. Nesting activity was analyzed using one-way Friedman-ANOVA followed by Dunn's multiple comparisons test. **c** Grooming scores of individual animals are shown for male (upper graph) and female (lower graph) mice as median and range (n = 21 per sex). Fluorescence suspension was applied 10 h after start of d.w. treatment to mouse skin. Upper x-axis indicates time points (h) during BL2 and after start of d.w. treatment (MEL). Lower x-axis shows time points (h) after administration of fluorescence suspension. Arrows indicate administration of fluorescence. Two-way ANOVA followed by Šídák's multiple comparisons test was performed. **d** Time (h) after application of fluorescence to achieve grooming score 5 is shown. Data are presented as median and range (n = 21 per sex). One-way ANOVA and Dunnett's multiple comparisons test shows significantly prolonged time to achieve score 5 for female mice under MEL d.w. treatment vs. BL2 in the second trial ($p = 0.0054$).
